# Finite element-based feasibility study on utilizing heat flux sensors for early detection of vascular graft infections

**DOI:** 10.1038/s41598-023-42259-y

**Published:** 2023-09-27

**Authors:** Signe Lin Kuei Vehusheia, Cosmin Roman, Rafael Sonderegger, Nikola Cesarovic, Christofer Hierold

**Affiliations:** 1 Department of Mechanical and Process Engineering, ETH Zurich, Zurich, Switzerland; 2 Department of Health Sciences and Technology, ETH Zurich, Zurich, Switzerland; 3 Department of Cardiothoracic and Vascular Surgery, German Heart Center Berlin, Berlin, Germany

**Keywords:** Fluid dynamics, Diagnostic markers, Infection

## Abstract

Aortic vascular graft infections have high morbidity and mortality rate, however, patients often do not show symptoms. Continuous implant surface monitoring will allow for early detection of infections on implant surfaces, which allows for antibiotic treatment prior to biofilm formation. We explore the possibility of using heat flux sensors mounted on an aortic vascular graft to sense the localized heat production at the onset of infectious growth. We apply Finite Element Model simulations to demonstrate changes of the heat transfer coefficient depending on different pulsatile flow parameters. We determine various differences, the main influence being the distance travelled from the inlet of the simulation with the highest heat transfer coefficient closest to the inlet and decreasing along the direction of travel of the fluid. The determined range of heat transfer coefficients of 200 to 4800 W/m^2^ was applied to a second simulation of the thermal environment of the implant. We determined the heat transfer efficiency of the aortic graft system depending on different graft materials and thicknesses. We are further able to determine that the early detection of infection is possible by comparing the simulated amount of heat flux produced locally with the resolution of a commercial heat flux sensor.

## Introduction

Implant infections are rare, however, with increasing number of cardiovascular disease patients receiving implants and treatment, an increasing number of individuals face complications such as infections^1^. Vascular grafts are used to overcome blockages and damage to the vascular walls. In the case of vascular grafts infections occur in 0.5–4% of implants^2^. However, in such cases the mortality ranges between 17 and 40% of the cases^3^. Upon bacterial adhesion from a source of contamination, bacterial growth is facilitated on the implant surface in relation to other locations in the body^4^. Fewer bacteria are necessary to infect a surgical site if an implant surface is present than without an implant surface^5^. Occasionally, the bacteria could form a biofilm of extracellular polymers^6^, which makes them become resistant to antibiotic treatments^7^. The current methods for infection detection are time consuming, expensive, and often inconclusive^8^, and only applied ensuing a suspected vascular graft infection^3^.

The approach of personalized, preventive medicine with a sensor for the early detection of infection has the potential to decrease the mortality rate and significantly improve the treatment of implant infections, as are currently the issues in vascular graft infection^9–11^. Various chemical and biochemical sensors have been investigated for infection detection (C-reactive protein^12–14^, lactate and glucose^15–17^, pH^18–21^), however, these sensors come with the limitations of drift and long-term stability of chemical and biochemical sensors in vivo (e.g. encapsulation due to foreign body response^9,22,23^). To combat these shortcomings alternative infectious markers such as temperature have been investigated for the potential detection of infection and inflammation^24,25^. Thermal power [W/m^3^] produced at infection sites areas is larger than the one of healthy tissues, as shown through their representation in previous simulation work^26–28^.

Our approach towards early detection of vascular graft infection is using an array of heat flux sensor embedded in the implant wall. The use of a heat flux sensor allows for the decoupling from globalized temperature fluctuations (fever, sport, and sleep) and focus on the localized heat production by infectious growth. Previous literature show that heat flux sensors embedded in microfluidic chips made of PDMS can detect the onset of bacterial growth from a heat source thermal density of 1707 W/m^3^^29^ which is in line with the early detection of bacterial growth at the onset of the exponential growth curve. With an approach of having a physical sensor (heat flux sensor) with the potential of long-term stability, we open the possibility of early detection and treatment of implant infections, drastically decreasing the mortality rate from between 17 and 40%^3^. In this case, antibiotics would still have the potential to be administered with success, prior to biofilm formation^11,30^.

**Figure 1:**
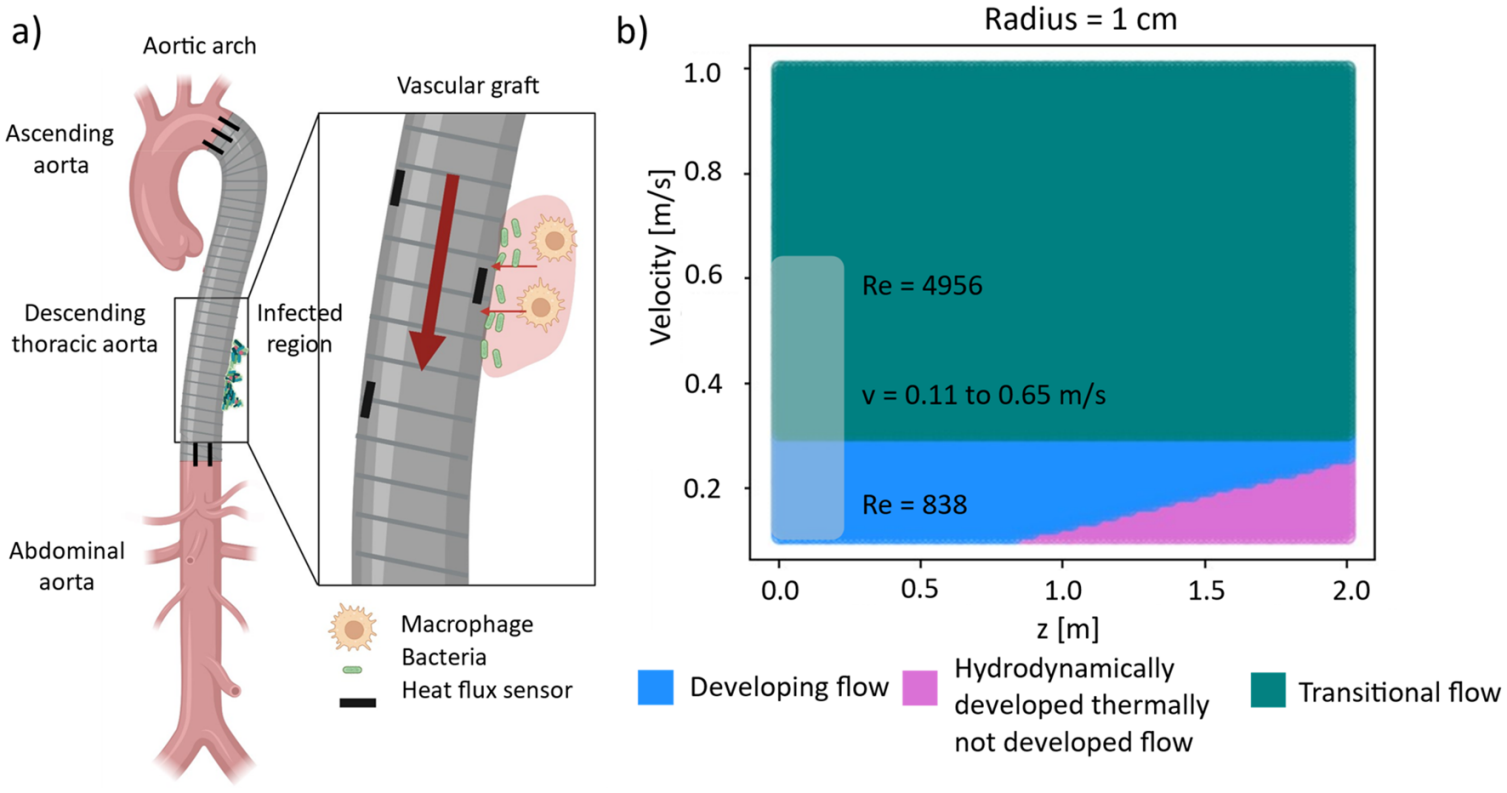
Heat transfer of environment of the infection detecting vascular graft and sketch overview over the 2D axisymmetric fluid dynamic regimes. (**a**) Sketch (created with BioRender.com) of the model system of a vascular graft with an implanted heat flux sensor and a growing infectious region. (**b**) The different flow regions for an inner radius of the aorta of 1 cm with varying velocity and distance travelled from the flow inlet. The physiological region of relevance is highlighted in white (0 < z < 20 cm and 0.11 < v < 0.65 m/s).

To determine the feasibility of the application of a heat flux sensor, the thermal environment of an infectious growth must be analyzed. In the case of the vascular graft, the localized heat produced by the infection can either go towards the tissue, or in the direction of the implant, embedded heat flux sensor and the blood flow (Fig. 1a). In this system, the blood flow effectively has the property of a heat drain, and the heat transfer in the direction of blood flow can be defined by the heat transfer coefficient. The influence of pulsatility in pipe flow on the heat transfer coefficient has previously been discussed in literature with varying results in different laminar flow regions (developing flow, hydrodynamically fully developed and thermally not developed flow, and fully developed flow)^31^. Kim et al.^32^ and Chattopadhyay et al.^33^ numerically investigated the influence of pulsatility on the heat transfer coefficient in the developing flow, and both did not find an impact of the pulsatility on the heat transfer coefficient. Habib et al.^34^ experimentally determined that the frequency of the pulsatility determines whether the heat transfer coefficient increases or decreases. An increase of 40% was determined for a specific set of frequencies whereas a decrease of 20% was found for another set of frequencies. In the hydrodynamically fully developed, and thermally developing region Hemida et al.^35^ found a up to a 6% decrease in the heat transfer coefficient, whereas Chattopadhyay et al.^33^ found no impact by the pulsatility, however, determined a large influence of the distance travelled from the inlet of the flow. In the fully developed flow region, both Yu et al.^36^, and Hemida et al.^35^ found no significant influence of pulsatility on the of heat transfer coefficient. Due to the experimental and numerical difficulty of turbulent flow, the studies in literature are limited. Zhao et al.^36^ investigated the influence of pulsatility in the fully turbulent flow region.

Most previous studies on pulsatility in pipe flow focus on the cooling or heating effects for larger scale processes, with only a few papers focusing on biofluid dynamic flow conditions. Craciunescu et al.^37^ investigated the influence of pulsatility on the temperature distribution in dog aorta and blood vessels, avoiding the low Reynolds number turbulent flow region by addressing the laminar regime. Most of these studies have focused on the laminar flow regimes, as turbulence still poses a theoretical difficulty.

We investigate forced convection single-phase pulsating fluid flow in a constrained environment in the low Reynolds number turbulent environment, comparing its influence on the pulsatile flow. Here, we present an FEM simulation of the influence of pulsatile flow on the heat transfer coefficient at the aortic wall. The model system we wish to investigate is the treatment of descending aortic disease using the replacement of aortic tissue with a vascular graft. The goal is to investigate the possibility of introducing heat flux sensors in the wall of these vascular graft implants for the potential early detection of infectious growth around the implant. In our simulation approach, we determine the range of heat transfer coefficients determined by the Womersley flow modelled. Further, we show that in the descending aorta, the flow regime is in the low Reynolds number turbulent regime, where the distance travelled from the inlet of the simulation is of more importance than the pulsatility. Moreover, by applying the range of heat transfer coefficients determined, we also determined the heat transfer efficiency of the vascular graft for a heat flux sensor in the implant wall. Furthermore, we investigated the measurable heat flux through the heat flux sensor from an infectious source with increasing heat density [W/m^3^], radius [mm], and distance [mm] to the sensor.

## Results

### Determination of heat transfer coefficient range in the descending aorta

#### Model system description of the heat transfer coefficient determination

Figure 1a shows the investigated region, namely the descending thoracic aorta, where the vascular graft infection detection system is investigated later. The infected area is expected to grow on the tissue side of the vascular graft as shown in the close-up in Fig. 1a. The heat transfer coefficient on the liquid side of the vascular graft is expected to depend on flow conditions, including flow rate, position along the aorta and flow pulsatility, and will determine the heat flux measured by the heat flux sensors^38^.

The radius of the descending aorta is around 1 cm^38^ and the radius of available grafts are also of similar size, therefore the radius of the simulation is also determined as 1 cm. The input velocity of blood flow ranges between 0.11 and 0.65 m/s^39^ as has previously been measured as the relevant blood flow velocity in the aorta. The length of the thoracic aorta has been determined to be between 33.2 and 37.3 cm, and the section that is the descending aorta (which is the part which we are interested in) has been determined to be between 20.8 and 22.5 cm literature^40^. We model a vascular graft in the region of the descending aorta, as this is the section of the aorta which is closest in geometry to our simulation model build. The flow region of relevance for the descending aorta assuming a straight cylinder and the described flow conditions, is in the developing flow region (${z<L}_{h},{L}_{th}$; hydrodynamic and thermal entry lengths). And the flow condition is in the transitional turbulent low Reynolds number, $Re$, flow regime ($2300<Re<8000$) as indicated in Fig. 1b (parameters defined and determined in Section 1 of the Supplementary Information (SI)).

**Figure 2:**
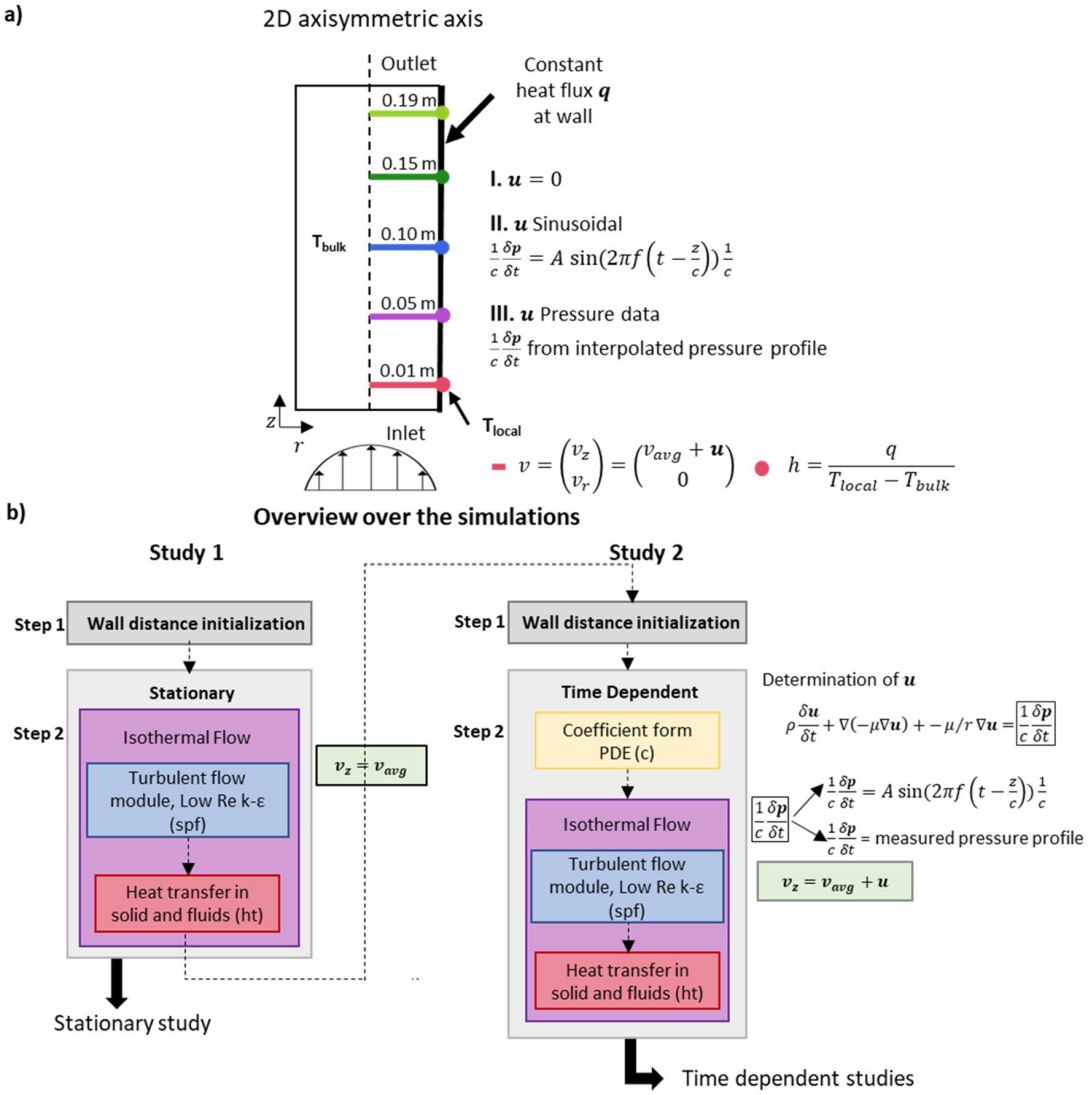
Schematic overview over the flow simulation—sketch and detailed simulation setup. (**a**) Sketch over the 2D axisymmetric simulation setup. The color code at the distances travelled, z, is used throughout the text for the simulation results. The input velocity ${\mathrm{v}}_{\mathrm{r}}=0$ and ${\mathrm{v}}_{\mathrm{z}}={\mathrm{v}}_{\mathrm{avg}}+\mathrm{u}$ are used at the inlet. The three different simulations are indicated by I, II, and III. as the three different values for $\mathrm{u}$; 0, sinusoidal, and measured blood pressure profile. The heat transfer coefficient is determined at the cylinder wall as $\mathrm{h}=\mathrm{q}/{(\mathrm{T}}_{\mathrm{local}}-{\mathrm{T}}_{\mathrm{bulk}})$, with $\mathrm{q}$ as the given constant heat flux input at the cylinder wall. (**b**) The simulation setup for both the stationary (study 1) and the time dependent simulation (study 2). Further described in detail in the methods section.

However, determining the heat transfer coefficient for flow conditions other than the fully developed laminar flow is a complex problem, which does not yield a closed form expression. Since the flow conditions we investigate lie within the developing flow region and the low Reynolds number turbulent flow regime, we require an appropriate numerical approximation such as Finite Element Modelling (FEM). COMSOL Multiphysics is utilized to model the coupled heat transfer and fluid dynamics for a 2D axisymmetric cylindrical aorta, for both stationary and pulsatile flow regimes, (Fig. 2a). Through FEM simulation, temperature and velocity profiles are solved for, allowing direct calculation of the heat transfer coefficient, $h$. As a result, the heat transfer coefficients are determined at the aortic wall for the different flow scenarios (Fig. 2b). From these investigations, as detailed in the following subsections, a range of heat transfer coefficients are extracted. They are further used in the 3D infection detection vascular graft system simulation to investigate heat flux measured by sensors upon infectious growth with the purpose of determining the feasibility of early detection of infection on vascular grafts (Dacron, PTFE, and ePTFE) using heat flux sensors.

#### ${{{v}}}_{{{z}}}={{{v}}}_{{{a}}{{v}}{{g}}}+{{u}}$, heat transfer coefficient determination with stationary velocity

**Figure 3:**
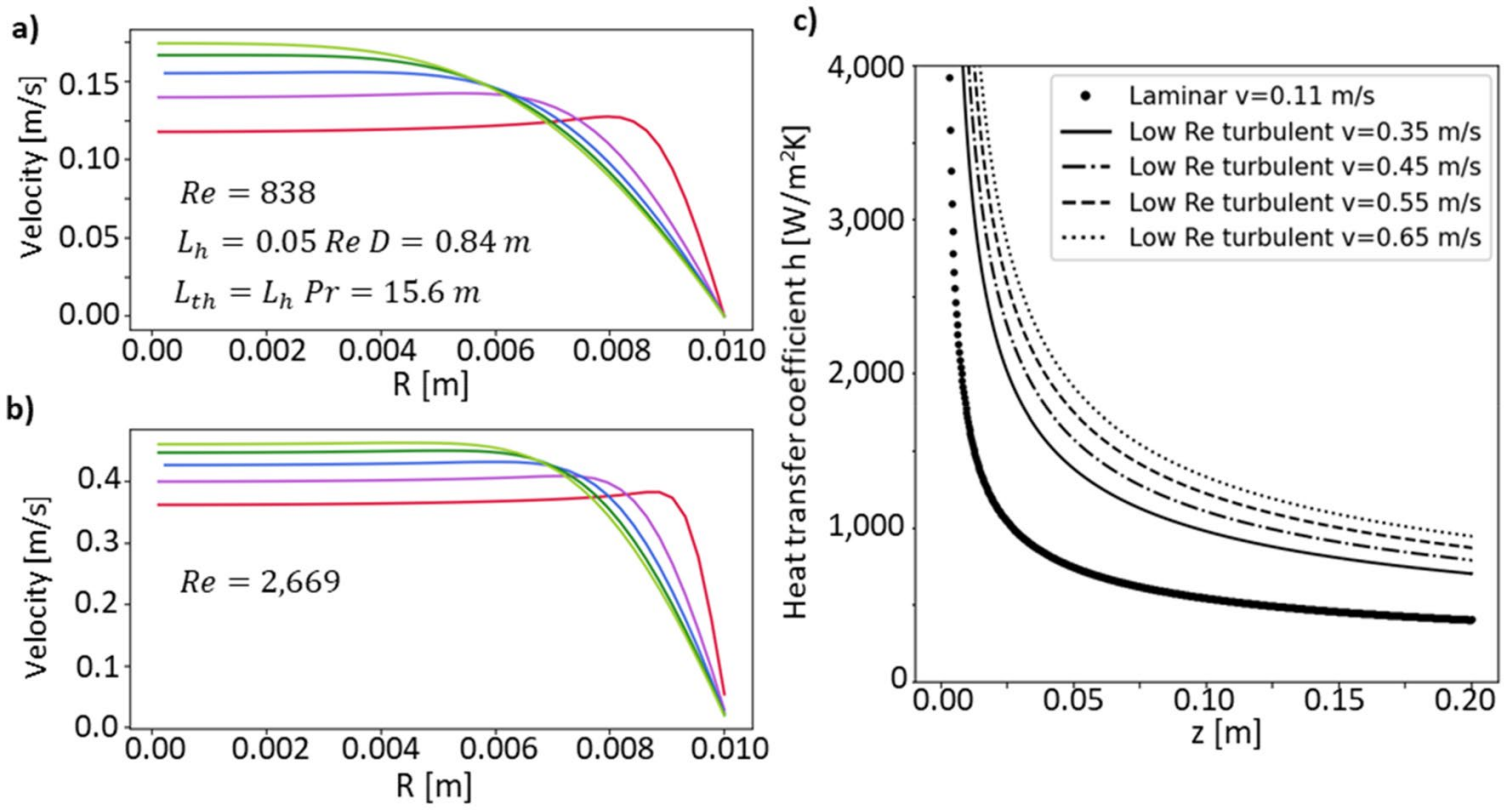
Velocity profiles at different points along the direction of flow in the laminar and low Reynolds number turbulent flow regions. (**a**, **b**) The velocity profile in the radial direction at different distances from the inlet (color code according to Fig. 2b). In (**a**), the input velocity is set to 0.11 m/s and the flow is fully laminar, whereas in (**b**) the velocity is set to 0.35 m/s and the flow is in the low Re turbulent flow region. The low turbulent Re flow flattens the velocity profile along the flow in z direction in comparison to the laminar flow regime, in a cylinder of same geometry. (**c**) The heat transfer coefficient at the aorta wall for different velocities for both laminar and low Reynolds number turbulent flow.

In the case of stationary velocity, $u=0$ (study 1 in Fig. 2b), we solved for the velocity profile and heat transfer coefficient, $h,$ using the isothermal flow Multiphysics model. Figure 3a and b show the velocity profiles in the radial direction at different distances, $z$, from the inlet (using the same color code as in Fig. 2a) for $v= 0.11 \mathrm{m}/\mathrm{s}$ in the laminar flow regime and $v= 0.35 \mathrm{m}/\mathrm{s}$ in the low Re turbulent flow regime. In the laminar flow region, $Re=838$ and ${L}_{h}=0.42 \mathrm{m}$, as shown in Fig. 3a, the development of the velocity profile along the length of travel converges towards the Hagen Poiseuille flow profile. In direct comparison, the low $Re$ turbulent flow, with $Re=2669$, the turbulence flattens the velocity profile and does not converge towards the Hagen Poiseuille flow profile.

For the subsequent comparison between $h$ with a stationary velocity and a pulsatile velocity, we determined $h$ along the aortic wall for a range of different stationary velocities (Fig. 3c). Our results are comparable to previous results shown in literature for pipe flow^33^, where it is shown that $h$ decreases along the distance travelled from the simulation inlet, $z$. We observe a steep decrease in $h$ between $v=0.00 \, {\mathrm{m}}$ and $v=0.05 \, {\mathrm{m}}$, whereas a gradual slope is found between $z=0.05\, {\mathrm{m}}$ and $z=0.20\, {\mathrm{m}}$. A sum of power law functions in the form of $h(z)=a{z}^{-\beta }+{cz}^{-\gamma }$ captures the $h(z)$ relationship in the low Re turbulent flow regime ($v=0.35, 0.45, 0.55, 0.65\, {\mathrm{m}}/{\mathrm{s}}$) well. Applying this fit in the low Re turbulence regime, we determined that $\beta =0.5$ and $\gamma =1.5$ with R^2^ values ranging from 0.96 to 1.00, as shown in Section 1 of the SI. Furthermore, we observe a linear relationship between the velocity and the fitting parameters $a$ and $c$. An increase in velocity increases the heat transfer coefficient. This suggests a predictability of $h$ along the z axis for low Re turbulent flow regime depending on the constant velocity applied.

In physiological conditions, the aortic radius slightly decreases along the direction of travel^38^, $z$. A decrease in the radius along the z axis increases $h$, thereby changing the decreasing slope of $h\left(z\right)$ observed in a system with constant radius. The tapered aortic simulations are investigated further in Section 3 of the SI.

#### ${{{v}}}_{{{z}}}={{{v}}}_{{{a}}{{v}}{{g}}}+{{u}}$, heat transfer coefficient determination with sinusoidal flow profile

**Figure 4:**
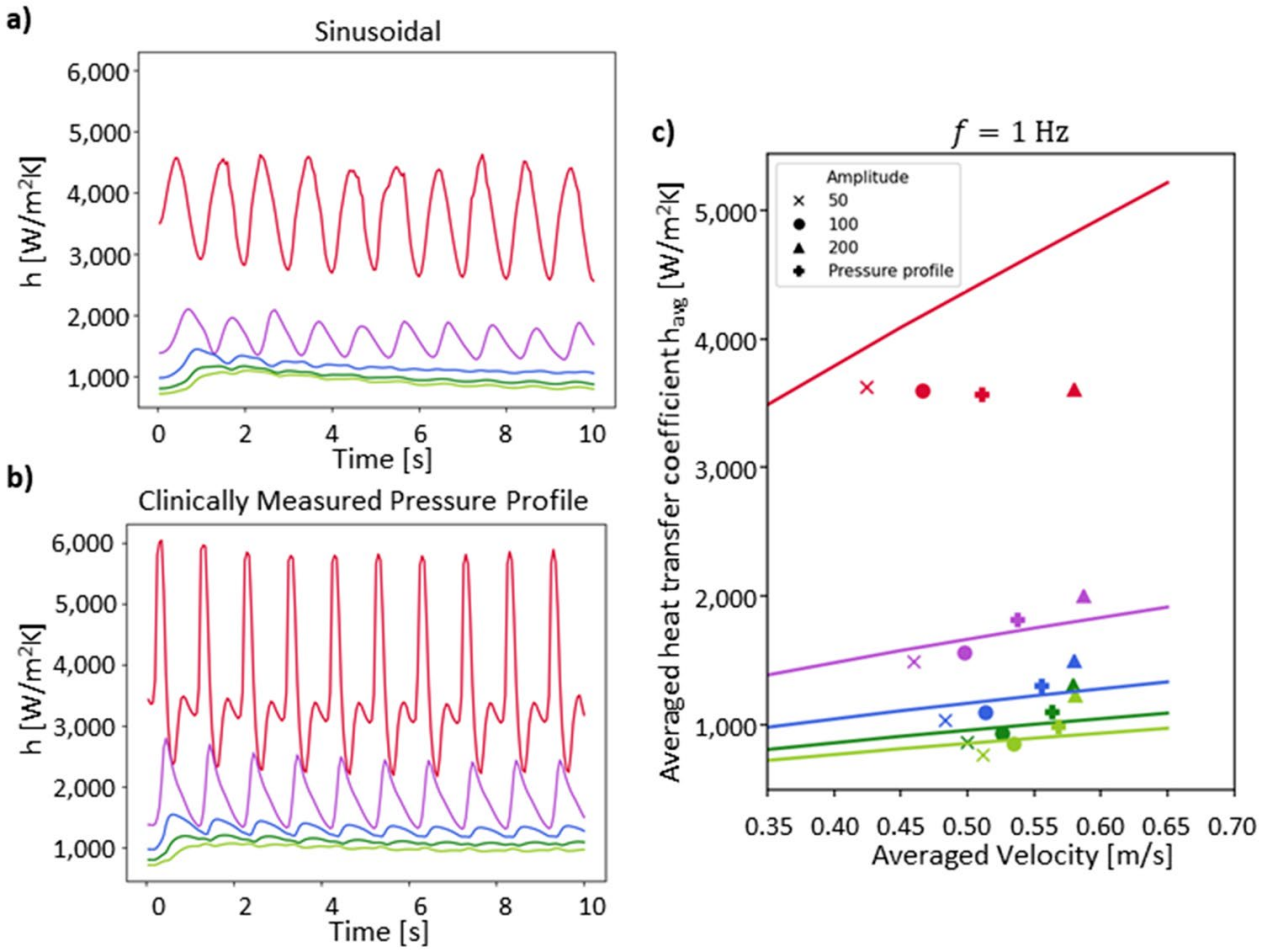
Pulsatile investigation of the time dependent determination of the heat transfer coefficient. (**a**, **b**) Heat transfer coefficients, $\mathrm{h}$, for sinusoidal (**a**) and aortic pressure measurement-based flow (**b**), respectively for $\mathrm{f}=1 \, {\mathrm{ Hz}}$ and $\mathrm{A}=100 \, {\mathrm{ Pa}}/{\mathrm{m}}$. Similar to the constant velocity simulation, $\mathrm{h}$ was determined by $\mathrm{h}=\mathrm{q}/({\mathrm{T}}_{\mathrm{local}}-{\mathrm{T}}_{\mathrm{bulk }})$ and plotted over time. The oscillation of the input velocity is reflected in the heat transfer coefficient. (**c**) Comparison between heat transfer coefficient for constant velocity (continuous lines) and a parametric sweep of the oscillatory flow (both sinusoidal and interpolated pressure profile). Shows the heat transfer coefficients for $\mathrm{f}=1 \, \mathrm{ Hz}$ in the sinusoidal parametric sweep. The heat transfer coefficient and the velocity are averaged over the last 5 s of the simulation.

We applied a pulsatile velocity element, $u$, by solving a coefficient for a partial differential equation as illustrated in Fig. 2b in study 2. Figure 4a shows the heat transfer coefficient along the aortic wall at increasing distances from the inlet, z, following the color code as shown in Fig. 2a. To investigate the influence of the pulsatility on $h$, the amplitude, $A$, and the frequency in a physiological region ($f=1, 2$) and 3 Hz were parametrically swept. Both these parameters change the source term, $\frac{1}{c}\frac{\delta P}{\delta t},$ in the partial differential equation solving for the pulsatile velocity element $u$. The Womersley numbers for blood at $f=1 \, {\mathrm{Hz}}$ correspond to $\alpha =15$. The values give an indication as to how the velocity profile develops over time in relation to the introduced pulsatility (full velocity profiles and further frequency variations are shown in Section 4 of the SI).

In comparison to the heat transfer coefficient $h,$ for constant velocities (continuously drawn line in Fig. 4c), the pulsatility both decreases and increases $h$ in the parametric sweep at $f=1 \, \mathrm{Hz}$ (full data sets shown in Section 4 of the SI). Heat transfer coefficient $h$*,* and the velocities, $v$, as solved for in the simulation (Fig. 4c) are averaged over the last $5 \, \mathrm{s}$ (of a $10 \, \mathrm{s}$ simulation). To study the influence of the pulsatility on the changes in $h$, the percentage change of $h$ at the same velocity for constant flow is determined. The change in $h$ upon pulsatility for $f=1 \, \mathrm{Hz}$ is determined from 25% under the constant velocity to 33% over. Averaging all the percentage changes, the overall difference between $h$ for pulsatile flow and constant flow is $-1\pm 16 \%$. For $f=2$ and $3 \, \mathrm{Hz}$, $h$ is consistently below that of constant velocity $h$ with a total average decrease of $-10\pm 3 \%$ and $-11\pm 2 \%$ respectively (as shown in Table T3 in the SI). All averaged values are in a similar range to what is found in literature of a decrease in $h$ of 6% upon flow pulsatiltiy in the laminar flow range^41^. An increase in the radius increases the heat transfer coefficient in the cylinder simulation model as discussed in Section 3 of the SI.

#### ${{{v}}}_{{{z}}}={{{v}}}_{{{a}}{{v}}{{g}}}+{{u}}$, heat transfer coefficient determination with an interpolated pressure profile

As blood does not flow with a sinusoidal profile, we interpolated and derived a previously measured aortic pressure profile $P$ from literature^42^ and applied a time derivative. We used this as the source term $\frac{\delta {\varvec{P}}}{\delta t}$ in the previously mentioned partial differential equation as shown in Fig. 2b study 2.

In contrast to the sinusoidal pulsatility we observe a general increase in the averaged pulsatile $h$ in comparison to constant velocity for $z$ between $0.02$ and $0.19 \, \mathrm{m}$ of $7\pm 2 \%$(total average of $1\pm 12 \%$). This is similar to the behavior of $h$ at a frequency of $1 \, \mathrm{Hz}$ and an amplitude of $A=200 \, \mathrm{Pa}/\mathrm{m}$. This is in line with the observation that the interpolated pressure profile has a frequency of $1 \, \mathrm{Hz}$, and a larger amplitude than the sinusoidal flow profile at $A=100 \, \mathrm{Pa}/\mathrm{m}$, as apparent when comparing Fig. 4a and b. Similar to the sinusoidal simulation ($1 \, \mathrm{Hz}$ with $A=200 \, \mathrm{Pa}/\mathrm{m}$) at $z=0.20 \, \mathrm{m}$, we observe a decrease in the averaged $h$ instead of an increase along the distance of travel. In literature, increases in $h$ have been experimentally measured in the turbulent flow region for pulsatile flow, however, at a significant larger $Re$ range than investigated here^43^. We find that the largest influence on the $h$ remains the distance travelled from the inlet of the simulation, $z$. A range of relevant heat transfer coefficients of the simulated aortic blood flow can thereby be conservatively determined from $200 \, \mathrm{W}/({\mathrm{m}}^{2}\mathrm{K})$ at $0.20 \, \mathrm{m}$ to $4800 \, \mathrm{W}/({\mathrm{m}}^{2}\mathrm{K})$ at $0.01 \, \mathrm{m}$, considering pulsatile flow.

### Infection detection vascular graft system simulations

**Figure 5:**
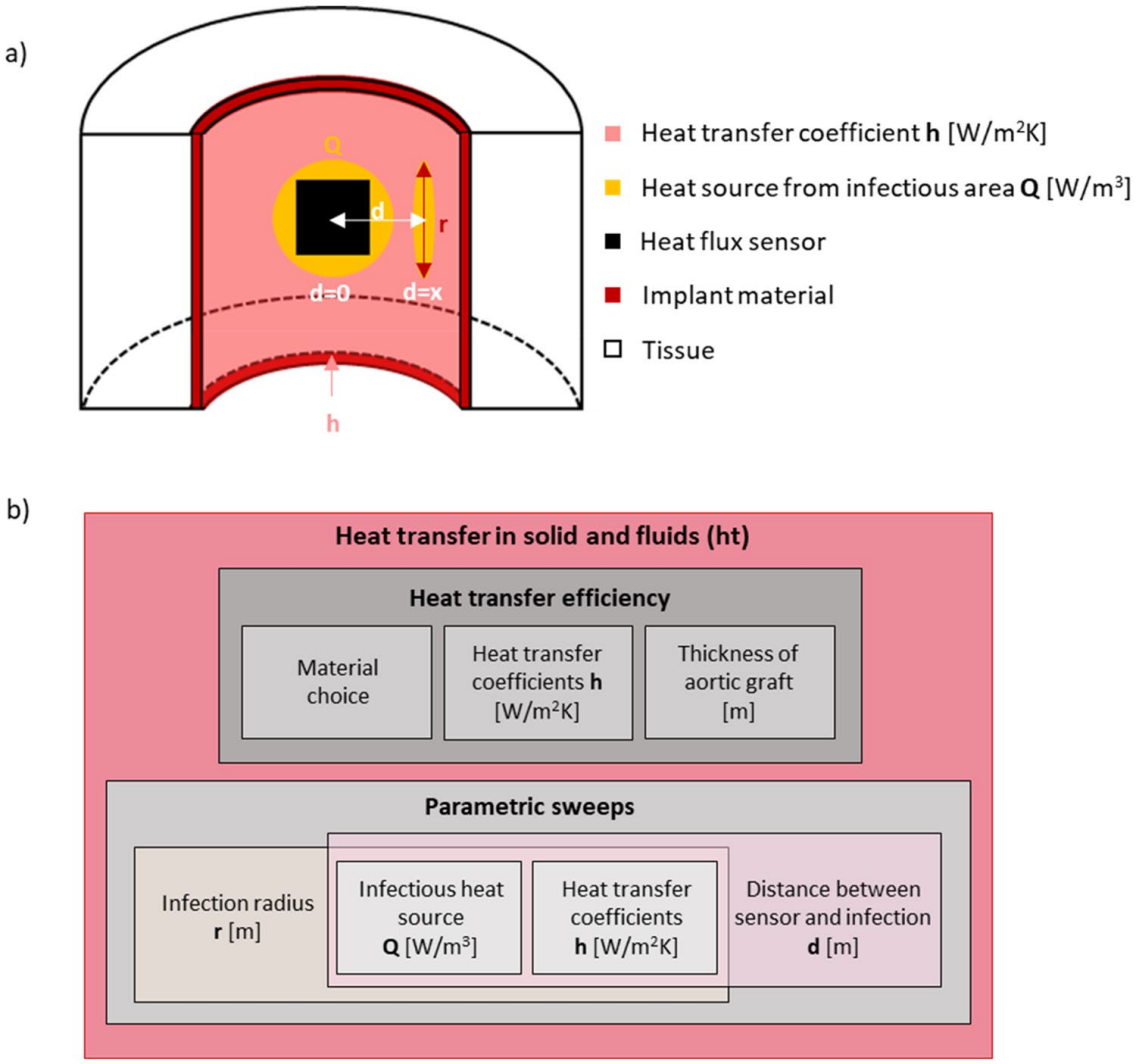
Simulation overview of the thermal simulation for the infection detection vascular graft system. (**a**) Schematic overview of the simulation structure for the aortic vascular graft. In the simulation design, the radius of the infection, the infectious heat source, the heat transfer coefficient, and the arc length (d) between the middle of the sensor to the middle of the infection are varied as shown in Section 8 of the SI. (**b**) Overview of the different simulations of the system. The heat transfer efficiency is determined with a parametric sweep of the heat transfer coefficients. A general parametric sweep over the heat transfer coefficients, infectious heat source, infectious radius, and the arc length (d) between the sensor and the infection is performed to determine at which point the heat flux through the sensor is above the resolution of a commercial heat flux sensor in the relevant dimensions.

**Figure 6:**
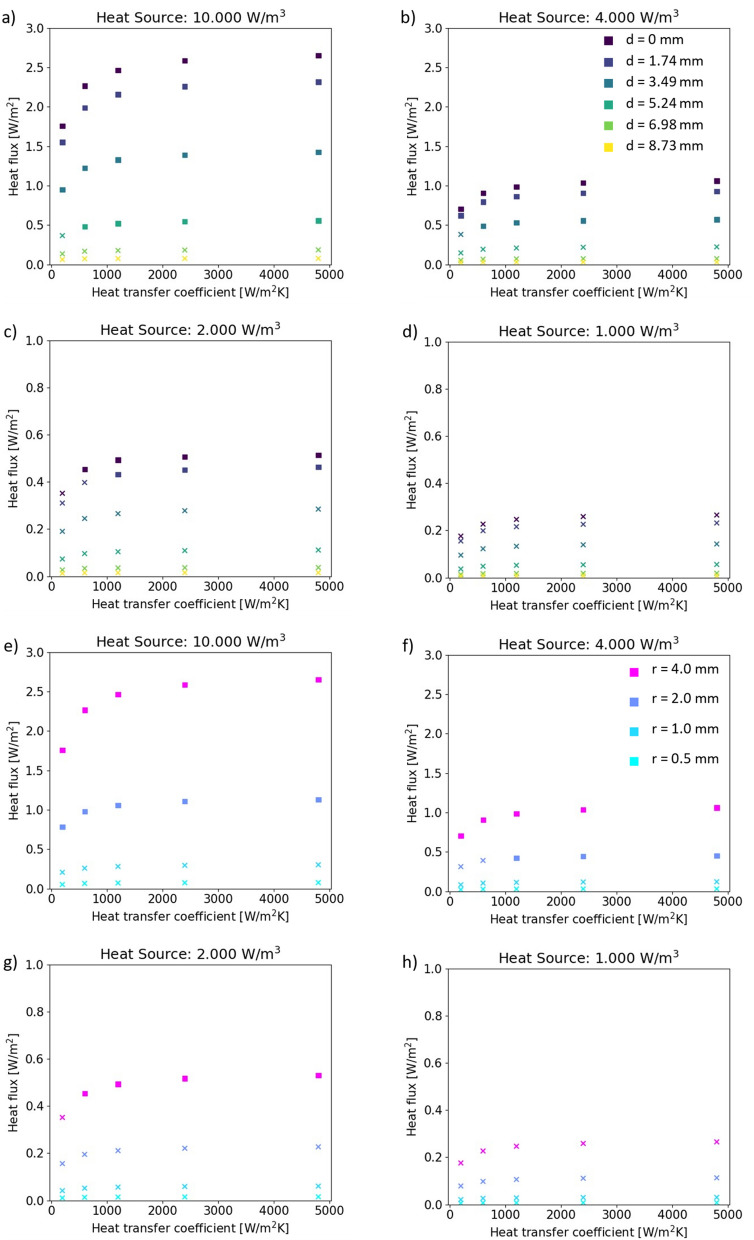
Parametric sweeps. (**a–d**) Variation in the arc length, d, from the center of the sensor to the center of the infection, and the corresponding heat flux measured through the heat flux sensor in the simulation. Data points above the resolution of 0.41 W/m^2^ are indicated as squares and blow as crosses (**e**–**h**) Variation in the radius of the heat source, r, and the corresponding heat flux measured through the sensor in the simulation. Data points above the resolution of 0.41 W/m^2^ are indicated as squares and below as crosses.

In the previous section, we studied the heat transfer coefficient under varying flow conditions assumptions. A conservative range of $h$ was determined between $200 \, \mathrm{W}/({\mathrm{m}}^{2}\mathrm{K})$ to $4800 \, \mathrm{W}/({\mathrm{m}}^{2}\mathrm{K}$), which covers any flow range from stationary to pulsatile flow, aortic positions from $0.01$ to $0.20 \, \mathrm{ m}$, and average velocities between $0.35$ to $0.60 \, \mathrm{m}/\mathrm{s}$, covering most physiological conditions. We apply this range of $h$ to model the thermal environment of the aorta using a 3D cylinder (as illustrated in Fig. 5a) to investigate the feasibility of an early onset infection detection system using a heat flux sensor. This model is intended to reflect the case of a resection of aortic tissue and replacement of with an aortic vascular graft. The heat flux flowing through the sensor in the case of infection is simulated by a circular infectious heat source along the circular wall of the graft (Fig. 5a) on which a series of parametric changes were investigated (see Table Section 5 in the SI). To determine the thermal environment of the for the 3D cylinder mimicking an aortic vascular graft implant, we varied a range of different materials (expanded polytetrafluoroethylene (ePTFE), polytetrafluoroethylene (PTFE), Dacron and as a comparison the typically used prototyping material polydimethylsiloxane (PDMS), and even aortic tissue were investigated for comparison). These different materials were swept over a range of thicknesses as shown in Section 6 of the SI. Further, we determine the heat flux through the modelled sensor with a parametric sweep of the infection radius, thickness, and location in relation to the sensor, as shown in Fig. 6 and specified in Section 6 of the SI. The referenced resolution of a commercial heat flux sensor of 0.41W/m^2^ is then compared to the calculated heat flux values for each parametric configuration. The maximal temperature increase was determined for the different thermal power densities.

#### Heat transfer efficiency of the vascular graft implant system

The heat transfer efficiency is defined as the percentage ratio between the measured heat flux through the sensor and the heat flux created by a defined boundary heat input. The measured average heat flux through the heat flux sensor is ${q}_{avg}=({q}_{front \, surface}-{q}_{back \, surface})/2$. Assuming a low temperature increase by the infectious heat source, the boundary conditions were set to body temperature, $310.15 \, \mathrm{ K}$ (as elaborated on in Section 8 of the SI). To determine fitting parameters for the investigation of the infection detection vascular graft system simulation we compared different materials such as ePTFE, PTFE, Dacron, PDMS, and varied its thicknesses, as shown in Fig. 5a. For further insight, we also simulated for the case of an aortic wall to compare the thermal environment of vascular grafts in contrast. The heat transfer efficiency increases with increasing thermal conductivity $k$ (${k}_{\mathrm{PDMS}}<{k}_{\mathrm{PTFE}}<{k}_{\mathrm{ePTFE}}<{k}_{\mathrm{Dacron}}$) and decreasing material thickness around the sensor. As PDMS has the lowest thermal conductivity and thereby the lowest heat transfer efficiency, it is continuously used as our model system moving forward as it represents a lower bound and a worst-case heat transfer scenario. The range of heat transfer efficiencies is between $43 \%$ at $200 \, \mathrm{W}/({\mathrm{m}}^{2}\mathrm{K})$ to $66 \%$ at $4.800 \, \mathrm{W}/({\mathrm{m}}^{2}\mathrm{K})$, for a PDMS thickness of $250 \, \upmu \mathrm{ m}$, as shown in Table T7 in the SI. The increase in heat transfer efficiency is large in the step between $200 \, \mathrm{W}/({\mathrm{m}}^{2}\mathrm{K})$ and $600 \, \mathrm{W}/({\mathrm{m}}^{2}\mathrm{K})$ which corresponds to a positional change of $15 \, \mathrm{cm}$ or above. As this change is mainly influenced by $z$, the change in the heat transfer efficiency at the different positions can be compensated by the information of the position of the sensor and the additional physiological input (velocity, frequency, and amplitude).

#### Parametric sweep of infection radius, heat source, distance, and system heat transfer coefficient

Upon infectious bacterial growth, the number of pathogens in the region increases over time. We can correlate the heat source and the number of bacteria in the infectious volumetric entity and investigate the estimated heat produced by a bacterial biofilm. To determine the thermal density of a biofilm we first determine the thermal density of a single *Escherichia coli (E. coli)* bacterium. The thermal power of a single *E. coli* bacterium is $3.5 \, \mathrm{pW}$^44^, and the volume of an *E. coli* cell is $1.49 \, \upmu {\mathrm{m}}^{3}$^45^. Thereby, the thermal power density of one bacterium can be estimated as $\mathrm{2,350,000} \, \mathrm{W}/{\mathrm{m}}^{3}$. A bacterial biofilm with a bacterial occupation of 15%^46^ thereby would have a power density of $\mathrm{350,000} \, \mathrm{W}/{\mathrm{m}}^{3}$. The developing bacterial population is simulated by thermal power values considered ($1000 (0.29 \%)$, $2000 (0.57 \%)$, $4000 (1.14 \%)$ and $\mathrm{10,000} \, \mathrm{W}/{\mathrm{m}}^{3} (2.86 \%)$). These values can be expressed as percentages of the estimated power density of a fully developed bacterial biofilm (as also shown in Table T8 in the SI), which would produce $\mathrm{350,000} \, \mathrm{W}/{\mathrm{m}}^{3}$.

In comparison to muscle, $684 \, \mathrm{W}/{\mathrm{m}}^{3}$^27^, or viscera $3852 \, \mathrm{W}/{\mathrm{m}}^{3}$, the calculated biofilm thermal power density is orders of magnitudes larger. This opens the possibility of localized early detection of infection as the infectious area is expected to locally produce more heat than its surroundings. However, the thermal power of the initial acute inflammation coupled with the intrinsic inflammatory response immediately after surgery has previously not been quantified in literature. A different temporal behavior is expected between the acute inflammation and a pathogenic infection, as the heat sources are different. With the acute inflammation it is the immune system of the body acting with increased metabolism for the foreign body response, whereas, as previously discussed, each pathogen contributes to the increase in localized thermal power while also activating the increased metabolism of different immune cells. Therefore, a differentiation between tissue, acute inflammation and pathogenic infection is to be expected in the use case.

With these considerations as a reference point, we determined a range of thermal power densities of the infection for the parametric sweep and can express them as the percentages of thermal power produced in a bacterial biofilm as shown in Section 8 in the SI. The heat transfer through the sensor is determined as the average normal conductive heat flux [$\mathrm{W}/{\mathrm{m}}^{2}$] on the surface of the sensor facing the infection and the surface facing away. The determined heat flux through the sensor is compared to the resolution of the example of a commercial heat flux sensor of $0.41 \, \mathrm{W}/{\mathrm{m}}^{2}$ as shown in Fig. 6.

An infection will not necessarily grow directly above the heat flux sensor. An increase of the distance (arc length, d as illustrated in Section 8 of the SI) between the infection and the sensor or a decrease in the radius of the infection decreases the detectability of the growing infection, as shown in Fig. 6. An infection growing directly above the sensor ($d=0 \, \mathrm{mm}$), can be detected at $2000 \, \mathrm{W}/{\mathrm{m}}^{3}$($0.57 \%$). The infection detection threshold (expressed in percentages of the fully developed bacterial biofilm) can be conservatively estimated as $\mathrm{10,000} \, \mathrm{W}/{\mathrm{m}}^{3}$ which is only $2.86 \%$ of the heat of a fully developed bacterial biofilm, as shown in Fig. 6a. In this case, the infection can be as far as $d=5.24 \, \mathrm{mm}$ away from the heat flux sensor and still be detected.

As shown in Fig. 6e–h and more clearly in Section 8 of the SI, a heat source of $2000 \, \mathrm{W}/{\mathrm{m}}^{3}$ $(0.57 \%$ heat of a biofilm of $\mathrm{350,000} \, \mathrm{W}/{\mathrm{m}}^{3}$) for a $4 \, \mathrm{mm}$ radius and $450 \, \upmu \mathrm{m}$ thickness increases the heat flux through the sensor above the resolution of a commercial heat flux sensor. On the other hand, an infectious radius of $2 \, \mathrm{mm}$ would be detectable only if the bacterial population exceeds $1.14 \% \left(4000 \, \mathrm{W}/{\mathrm{m}}^{3}\right)$ of a biofilm.

The increase in temperature with a heat source of $2000 \, \mathrm{W}/{\mathrm{m}}^{3}$ and an infection radius of 4 mm ranges between $0.0025$ and $0.0030 \, \mathrm{K}$ at the location of the sensor for the whole range of determined heat transfer coefficients of $200 \, \mathrm{W}/{\mathrm{m}}^{2}\mathrm{K}$ to K $4800 \, \mathrm{W}/{\mathrm{m}}^{2}\mathrm{K}$ (as shown in Section 7 in the SI).

## Discussion

For the potential application of a heat flux sensor as an infection sensing component of a vascular graft in the case of aortic tissue replacement, the thermal environment was studied using two different simulation systems. The 2D FEM simulation determined a range of heat transfer coefficients in an aortic vascular graft dependent on the flow conditions and distance from the inlet of the considered and simulated region. We introduced two different types of pulsatility by the pressure profile; sinusoidal and interpolated from a measured pressure profile from literature to investigate the effects on $h.$ We determined that for the sinusoidal pulsatile flow $h$ is dependent on both the frequency and the amplitude for $z>0.01 \, \mathrm{m }.$ Regarding $h$, the closest matching sinusoidal input to the interpolated pressure profile of a patient is for the combination of $f=1 \, \mathrm{ Hz}$ and $A=200 \, \mathrm{Pa}/\mathrm{m}$. Both approaches showed increase in $h$ with pulsatility. For frequencies $f=1$ and $2 \, \mathrm{Hz}$, $h$ is decreased upon pulsatile flow for all amplitude variations with averages of $-10\pm 3 \%$ and $-11\pm 2 \%$ respectively. However, the largest influencing factor on $h$ is the distance travelled from the inlet of the simulation, $z$, as previously also found as a result in literature for laminar flow^33^.

Via a 3D simulation of the heat transfer of the infection detection vascular graft system, we determined the parametric range for the infection distance, size, and heat density at which the heat flux produced by the bacterial population exceeds the resolution of the heat flux sensor. An infectious area with a radius of $2 \, \mathrm{mm}$ and a thickness of $450 \, \upmu \mathrm{m}$ is above the commercial sensor resolution with a heat source of $2000 \, \mathrm{W}/{\mathrm{m}}^{3}$ (corresponds to $0.57 \%$ of the thermal power of a biofilm as described in Section 8 in the SI) and can be detected with a heat transfer coefficient of $1200 \, \mathrm{W}/{\mathrm{m}}^{2}\mathrm{K}$. We have previously shown in an experimental setup with heat flux sensor embedded in PDMS in a microfluidic chip that a heat source of $1707 \, \mathrm{W}/{\mathrm{m}}^{3}$ is detectable with a commercial heat flux sensor^29^. In that setup, two heat flux sensors were used to measure the differentially compensated heat signal of bacterial growth through common mode rejection, from the onset throughout the exponential bacterial growth in an in vitro microfluidic chip^29^.

As shown in Fig. 1a, we envision a system of multiple heat flux sensors placed in the vascular graft prosthesis, preferably in an array/mesh. This would allow for differential measurements between a sensor, above which there is an infection growing locally, and a sensor, above which there is no infection growing, essentially acting as common mode cancellation of the surrounding thermal fluctuations. This is based on the same principle as previously investigated in literature using two heat flux sensors measuring the onset of exponential bacterial growth in a PDMS based microfluidic chip^29^. In order to ensure a 2.86% infection detection threshold in relation to the fully developed biofilm, we suggest a hexagonal lattice of heat flux sensors with a lattice constant of placement of the heat flux sensors with a spacing of $\sqrt{3} \times 5.24 \, \mathrm{mm}$ over the surface of the graft. A 20 cm graft then needs 177 sensors to cover the surface. Relaxing the threshold for the onset of infection to $14.29 \%$ ($\mathrm{50,000} \, \mathrm{W}/{\mathrm{m}}^{3}$ as shown in Section 9 in the SI), we can decrease the number of sensors to 100 with a spacing of $\sqrt{3} \times 6.98 \, \mathrm{mm}$. There is a natural tradeoff between the determined infection detection threshold and the number of sensors needed for the coverage. An important body of future work would consist of the system integration aspects and clinical implementation. While wiring that many sensors may pose a challenge, it still seems feasible with the current state of sensor technology, in particular if we consider that the proposed heat flux sensors are passive sensors: they create an output voltage proportional to the heat flux without any support circuits needed for the operation of the sensor.

An increase in temperature in the body could be caused by the daily rhythm of the body (sport, sleep or even fever), weather, and activity—the temperature data therefore can be difficult to interpret. However, a localized change in the heat flux between a localized growing infection and a delocalized temperature source (blood) could give a clear indication of a growing infection. The simulated temperature increase (of $0.0025$ and $0.0030 \, \mathrm{K})$ at the onset of infection is around 30 times smaller than the resolution of most commercially available temperature sensors (estimated at $0.1 \, \mathrm{K}$). This showcases the benefits of utilizing differentially measuring heat flux sensors instead of temperature sensors. Nonetheless, the actual integration of the heat flux sensors in a vascular graft has yet to be developed and investigated in practice. Those investigations could clarify the relevance of the positioning and accuracy of the heat flux sensors. Furthermore, the compliance mismatch between the vascular graft material and the heat flux sensor should be considered in future studies as well.

The results of this study show the possibility of using an array of heat flux sensors on a vascular graft to allow for the early detection of infection. Future work includes investigating the influence of the initial curvature of the aortic arch on the heat transfer. Furthermore, aortic distensibility, branching and further physiological details (as can be taken from CT or MRI scans) could also influence the heat transfer coefficient and should be included in future work to present results that are closer to real-world situations. Nevertheless, the range of heat transfer coefficients studied here is broad which covers a broad set of realistic flow conditions. Furthermore, the choice of the low thermal conductivity of PDMS material gives a conservative estimation of the detection capabilities of the simulated system. These simulation results show the feasibility and potential of heat flux sensors for the early detection of infection. This approach could complement already existing systems such as blood cultures, surveillance CT scans and tagged white blood cells for the further improvement of patient treatment. In addition, our approach allows for a shift from late infection detection, as is currently the case, to continuous monitoring of infection growth, which opens new ways for improving diagnosis and preventive care.

## Methods and models

### Flow regions and thermal theory 2D axisymmetric system

The flow region is defined by the Reynolds number $Re=\rho vD/\mu$, with $\rho$ as the density, $v$ as the velocity, $D$ as the diameter, and $\mu$ as the dynamic viscosity. The laminar region can be defined by $Re<2300$. Further categorization of the laminar flow regions can be defined using the hydrodynamic entry length ${L}_{h}=0.05 Re D$ and thermal entry length ${L}_{th}={L}_{h}Pr$, where the Prandtl number is defined as $Pr={c}_{p}\mu /k$, with ${c}_{p}$ as the specific heat capacity, and $k$ as the thermal conductivity. Developing flow is defined as the region where ${z<L}_{h},{L}_{th}$, hydrodynamically developed but thermal not developed flow is defined as ${L}_{h}<z<{L}_{th}$, and fully developed flow is defined as $z>{L}_{h},{L}_{th}$. The transition to turbulence is defined by an intermediate region ${2300}<Re<8000$ called the transitional flow region, with a transition to turbulent flow at $Re>8000$. In the case of blood flow, the fluid velocity, pipe radius, and distance from flow source have a strong influence of the flow region. The additional pulsatility of blood flow is described by the Womersley number $\alpha =r\sqrt{2\pi f\rho /\mu }$, with $f$ as the frequency, $r$ as the system radius, $\rho$ as the density, and $\mu$ as the dynamic viscosity. $\alpha <1$ allows for the velocity profile to develop within the time of the frequency and $\alpha >10$ creates a flat velocity profile.

### Geometric model

In the 2D axisymmetric simulation, the descending aorta is simulated as a rectangular axisymmetric 2D model with a radius of 1 cm and a length of 20 cm using COMSOL Multiphysics 6.0 (COMSOL Inc., Sweden). We assumed no Windkessel effect, blood behaving as a Newtonian fluid, and no branching of the aorta. A decrease of the radius was investigated along the line of traveling, z.

In the 3D infection detection vascular graft simulation, the aortic structure was created by 3 cylinders (shown in Fig. S9 in the SI). The first cylinder represents the tissue with a 2 cm radius, the second cylinder represents the outer radius of the vascular graft with a radius of 1.105 cm, and the third cylinder represents the inner radius of the aorta with an inner radius of 1 cm. The infection was modelled by two separate cylinders, one in parallel with the cylinders of the aortic structure representing the thickness of the infection of a constant of 450 μm, one at an 90° angle to the other cylinders with a variable radius with varying position of the cylinder in relation to the sensor.

### Flow model

We approached the system in COMSOL as a 2D axisymmetric rectangle assuming blood to be a Newtonian fluid, we neglected the Windkessel effect, we assumed that there is no change in the radius along the fluid flow, and that there was no branching of the aorta in the region.

The simulation of the stationary system was first initialized using a wall distance initialization for the low-Re k–ε turbulent flow module. Using this solution, a stationary simulation with the turbulent flow low-Re k-ε module (spf) was used together with the heat transfer in solid and fluids module (ht) with the isothermal flow (Multiphysics). For the time dependent studies, the initial solution is set as the stationary study solution, and a further wall distance initialization is performed prior to the time dependent investigation. Prior to the partial differential equation solving for the time dependent oscillating component of the velocity, $u$, Isothermal Flow (low-Re $k-\varepsilon$ turbulent flow module and heat transfer in solid and fluids module (ht)), a wall distance initialization is performed again. Following this, a coefficient form PDE (c) is used to solve for the oscillating flow. Inserting the input of ${\mathrm{v}}_{\mathrm{z}}={\mathrm{v}}_{\mathrm{avg}}+\mathrm{u}$ for the flow, the system is solved using the Turbulent flow module (spf) and its influence on the heat transfer is determined using the isothermal flow Multiphysics module.

The input velocity $${v}_{z}={v}_{avg}+u$$ in the axial direction was spilt in two components, the average velocity $${v}_{avg}$$ (always set to 0.35 m/s for pulsatile flow simulations) and the pulsating velocity $$u$$. Three different inputs for the pulsating velocities $$u$$ are used to simulate constant velocity ($$u=0)$$, sinusoidal oscillation and a time dependent velocity profile based on physiological blood pressure measurement.

The oscillating part of the axial velocity $u$ of the axial velocity ${v}_{z}={v}_{avg}+u$ is solved by a partial differential equation (PDE) using the coefficient form PDE in COMSOL and expressed as:

$$\rho \frac{\delta {\varvec{u}}}{\delta t}+\nabla \left(-\mu \nabla {\varvec{u}}\right)-\mu /r\nabla \mathbf{u}=\frac{1}{c}\frac{\delta {\varvec{P}}}{\delta t}$$

The source term for the sinusoidal case is set to be:

$$\frac{1}{c}\frac{\delta P}{\delta t}=A \mathrm{sin}\left(2\pi f\left(t-\frac{z}{c}\right)\right)\frac{1}{c}$$

where $P$ is the pressure, $c$ is the pulse wave velocity of $8.8 \frac{\mathrm{m}}{\mathrm{s}}$, $A [\mathrm{Pa}/\mathrm{m}]$ as the amplitude, and $f [\mathrm{Hz}]$ is the frequency.

The source term of the interpolated pressure profile is set to be:

$$\frac{1}{c}\frac{\delta P}{\delta t}$$

where the pressure profile $P$ is interpolated from literature values^42^.

### Partial differential equation

The oscillating part of the velocity, $u$, is determined by using the Coefficient Form PDE (c) module, where the generalized equation is:

$${e}_{a}\frac{{\partial }^{2}u}{\partial {t}^{2}}+{d}_{a}\frac{\partial u}{\partial t}+\nabla \left(-c\nabla u-\alpha u+\gamma \right)+\beta \nabla u+au=f$$

With ${e}_{a}=0$ as the absorption coefficient, ${d}_{a}={\rho }_{b}$ as the damping coefficient, $c={\mu }_{b}$ as the diffusion coefficient, $\alpha =0$ as the conservative flux convection coefficient, $\gamma =0$ as the conservative flux source, $\beta =\frac{{\mu }_{b}}{r}$ as the convection coefficient, $a=0$ for the adsorption coefficient, and $f=\frac{1}{c}\frac{\partial P}{\partial t}$ as the source term. This yields:

$$\frac{\delta {\varvec{u}}}{\delta t}+\nabla \left(-\mu \nabla {\varvec{u}}\right)+-\mu /r\nabla \mathbf{u}=\frac{1}{c}\frac{\delta {\varvec{P}}}{\delta t}$$

where different source terms are given: $\frac{1}{c}\frac{\delta P}{\delta t}=A \mathrm{sin}(2\pi f\left(t-\frac{z}{c}\right))\frac{1}{c}$ and $\frac{\delta {\varvec{P}}}{\delta t}$ from interpolated measured pressure data from the aorta. The Dirichlet Boundary Condition was applied to apply the no slip wall condition for the oscillating velocity.

### Thermal model

In the 2D axisymmetric simulation, the Heat Transfer in Solids and Fluids (ht) is used for the fluid. In the stationary study the heat transfer equation used is $\rho {c}_{p}{\varvec{v}}\nabla T+\nabla {\varvec{q}}=Q+{Q}_{ted}$, with ${\varvec{q}}=-k\nabla T$ and in the time dependent case $\rho {c}_{p}\frac{\partial T}{\partial t}+\rho {c}_{p}{\varvec{v}}\nabla T+\nabla {\varvec{q}}=Q+{Q}_{ted}$, with ${\varvec{q}}=-k\nabla T$. A general inward heat flux of ${q}_{in}=200 \frac{\mathrm{W}}{{\mathrm{m}}^{2}}$ is applied at the boundary of $r=0.01 \mathrm{m}$.

The heat transfer coefficient at the cylinder wall is determined as:

$$h=\frac{q}{{T}_{local}-{T}_{bulk}}$$

with the given constant heat flux at the wall $q$, the local temperature at the wall ${T}_{local}$ and the bulk temperature:

$${T}_{bulk }=\frac{{\int }_{0}^{r/2}2\pi u\left(r,z\right) T\left(r,z\right)dr}{{\int }_{0}^{r/2}2\pi u\left(r,z\right)dr}$$

Both study 1 and study 2 are initialized using the wall distance initialization, whereafter the isothermal flow module is used to solve for the turbulent flow in the low Re flow region in combination with the heat transfer.

In the 3D simulation, the Heat Transfer in Solids (ht) is used for the whole geometry. The boundary condition at the inner radius is the heat transfer coefficient in the range between 200 and 4800 W/m^2^K, and the rest of the boundaries are set to 37 °C due to the small temperature increase of the tissue with the heat source as shown in Section 7 in the SI. A volumetric heat source [W/m^3^] is used for the infection. The vascular graft is modelled with PDMS to give a lower bound of the heat transfer efficiency in the simulations. The heat flux at the sensor was determined as an average of the heat fluxes on both sides. The commercial heat flux sensor resolution of 0.41 W/m^2^ to which the thermal measurements are compared to, is based on the gSKIN® XM 27 9C sensor from greenTEG AG.

### Supplementary Information

Supplementary Information.

## Data Availability

All data generated and analyzed during this study are available from the corresponding author following a request.
