# Supplementary material for: Finite element-based feasibility study on utilizing heat flux sensors for early detection of vascular graft infections
Source: Sci Rep. 2023 Sep 27;13:16198. doi: 10.1038/s41598-023-42259-y (PMC10533875; doi:10.1038/s41598-023-42259-y)
Supplement: Supplementary file 1 — Supplementary Information. [file 41598_2023_42259_MOESM1_ESM.docx]

**Supplementary Materials**

**1. Overview over the flow regions**


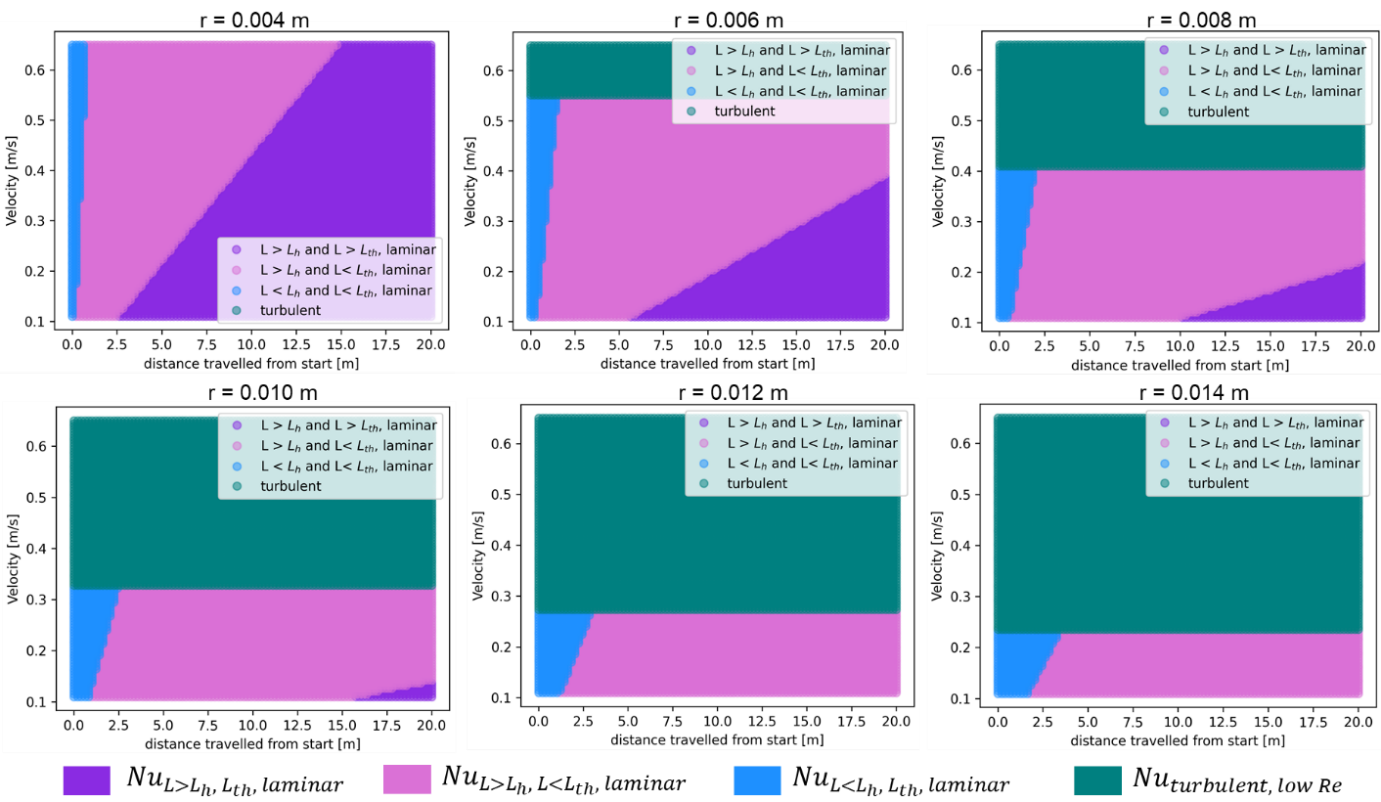


***Figure S1:*** *The variations of the flow regions as an expansion of Fig. 1 b). The flow regions are shown for an array of different aortic vascular graft radii. With an increasing radius, the turbulent region increases.*

| Velocity [m/s] | Reynolds number Re | Hydrodynamic entrance length $L_{h}$ [m] | Thermodynamic entrance length $L_{th}$ [m] |
| --- | --- | --- | --- |
| 0.11 | 838 | 0.42 | 7.81 |
| 0.35 | 2,669 | 1.33 | 24.9 |
| 0.65 | 4,957 | 2.48 | 46.2 |

***Table T1*** *The Reynolds number, hydrodynamic entrance length, and thermal entrance length for blood with a radius of 1 cm.*

The values below assume a radius of $r=0.01 m$ ($D=2r$ and $A=\pi r^{2}$), velocity of $v=0.35 m/s$ ($Q=vA$), density $\rho=1060 kg/m^{3}$, dynamic viscosity $\mu=0.00278 Pa s$, and thermal conductivity of $k=0.54 W/mK$. The material properties are that of blood at 37°C.

The Reynolds number, $Re$, for pipe flow:

$$Re=\frac{\rho QD}{\mu A}=2,669$$

With $\rho$ as the density [kg/m^3^], $Q$ as the flow rate [m^3^/s], $D$ as the hydraulic diameter [m], $\mu$ as the dynamic viscosity [Pa s], and $A$ as the area or the pipe cross section [m^2^]. $Re$ defines the transition between laminar and turbulent flow.

The hydrodynamic entrance length $L_{h}$ ^1^:

$$L_{h}=0.05ReD=1.33 m$$

The thermodynamic entrance length $L_{th}$ ^1^:

$$L_{th}=0.05ReDPr= 24.95 m$$

With $Pr=c_{p}\mu/k$ as the Prandtl number with $c_{p}$ [J/kgK] as the specific heat capacity and $k$ [W/mK] as the thermal conductivity. In our case for $Pr=18.6$ the hydrodynamic entry length is

For the fully developed laminar flow with a constant heat flux source, the heat transfer coefficient, $h=Nuk/D,$ is defined by^1^:

$$Nu=\frac{hD}{k}=4.36$$

In the case where the flow is thermally undeveloped $z<L_{th}$ and hydrodynamically undeveloped $z{<L}_{h}$ $Nu$ can be defined as ^1^:

$$Nu=4.36+\frac{0.065\left( \frac{D}{z} \right)RePr}{1+0.04(\left( \frac{D}{z} \right)Re{Pr)}^{2/3}}$$

In the case of thermally developed $z>L_{th}$ and hydrodynamically undeveloped $z{<L}_{h}$ $Nu$ can be defined as ^2^:

$$Nu=1.62{(RePr\frac{D}{z})}^{1/3}$$

In the case of low Re turbulent flow, $2400<Re<8000$ $Nu$ can be defined as ^2^:

$$Nu=0.037({Re}^{\frac{3}{4}}-180){Pr}^{0.42}(1+{(\frac{D}{z})}^{\frac{2}{3}})$$

These are the defining heat transfer coefficients for the different flow regions shown in Fig. S1.

**2. Parametric fitting of the model for different velocities**


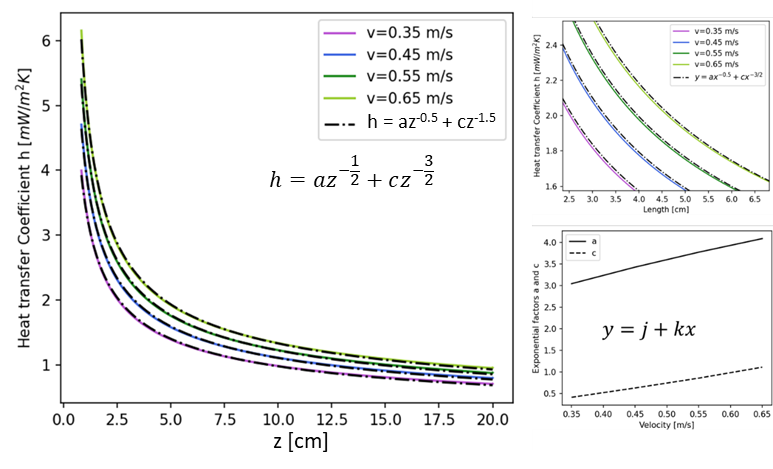


***Figure S2: Power law fit of the heat transfer coefficient along the aortic wall in the z direction.***

***Table T2: R^2^ values of the power law fit.***

| Velocity | 0.35 m/s | 0.45 m/s | 0.55 m/s | 0.65 m/s |
| --- | --- | --- | --- | --- |
| R^2^ | 0.9999983 | 0.9999989 | 0.9999991 | 0.96141257 |

The 2D axisymmetric FEM simulation for the case of stationary velocity allows for the extraction of the heat transfer along the aortic wall. Two power functions with different power exponents of -0.5 and -1.5 were used to fit a and c in the function. The R^2^ values are over 0.95 for all fits. Plotting a and c against the velocity used, a linear relationship becomes apparent, suggesting a predictability of the heat transfer coefficient in the low Re turbulent flow regime.

**3. Variation of the heat transfer coefficient depending on changes in radius**


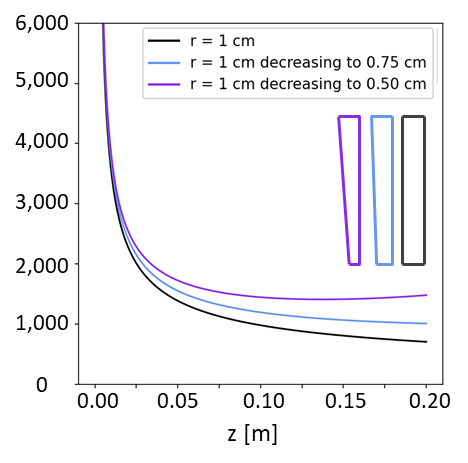


***Figure S3 Influence of radius variations on the heat transfer coefficient.*** *Shows the heat transfer coefficient with decreasing radius along the distance of travel.*

The heat transfer coefficient increases with decreasing diameter of the 2D axisymmetric cylinder. As expected, the heat transfer coefficient is similar to each other at the beginning. Upon further distance to the inlet, the heat transfer coefficient of the two cylinders with decreasing radius increase in relation to the constant radius simulation as also expected by the calculations for the heat transfer coefficient.


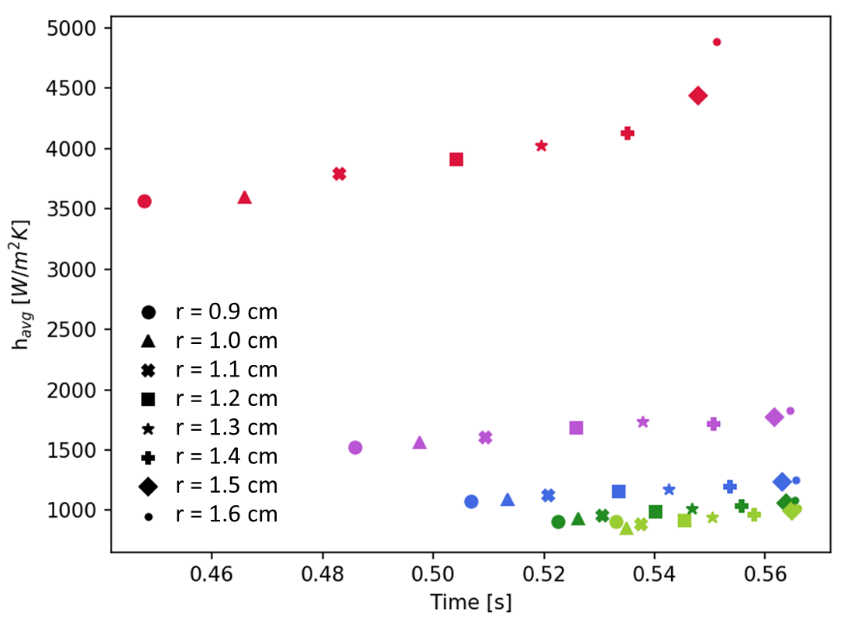


***Figure S4 Influence of radius variations on the heat transfer coefficient.*** *Shows the heat transfer coefficient with decreasing radius along the distance of travel. The color code follows the determined color code of Fig. 2 a) of different positions along the vascular graft, z as also used in Fig. 4. The heat transfer coefficients and the velocity were averaged over the last 5 seconds of the simulation.*

With an increasing radius of the aortic system, the averaged heat transfer coefficient increases. The broad range of radii chosen reflect the range of descending vascular graft diameter of d = 18 mm to 32 mm used in implants as reported in literature for descending vascular grafts ^3^. The increase is more significant at the inlet of the cylinder with a decrease in the influence of changes in the radius with increasing distance travelled from the inlet. An increase in the radius, as already shown in Fig. S1 and also according to the equations in literature, increases the turbulent flow regions within the cylinder.

**4. Parametric sweep of the sinusoidal input for the time dependent study**

The Womersley number can be defined as $\alpha=r\sqrt{\omega\rho/\mu}$ with $r$ as the cylinder radius (1 cm), $\omega$ as the angular frequency ($2\pi f, f=1 \mathrm{Hz}$ or $f=2 \mathrm{Hz}$ or $f=3 \mathrm{Hz}$), $\rho$ as the density ($1,059 kg/m^{3}$) and $\mu$ ($0.00278 Pa s$) as the dynamic viscosity ^4^. The Womersley number gives an indication between the shear force and the oscillatory inertial force, and for a large $\alpha$ ($\alpha>10)$ the velocity profile is plug-like, whereas for a low $\alpha$ ($\alpha<1)$ a parabolic velocity profile develops.


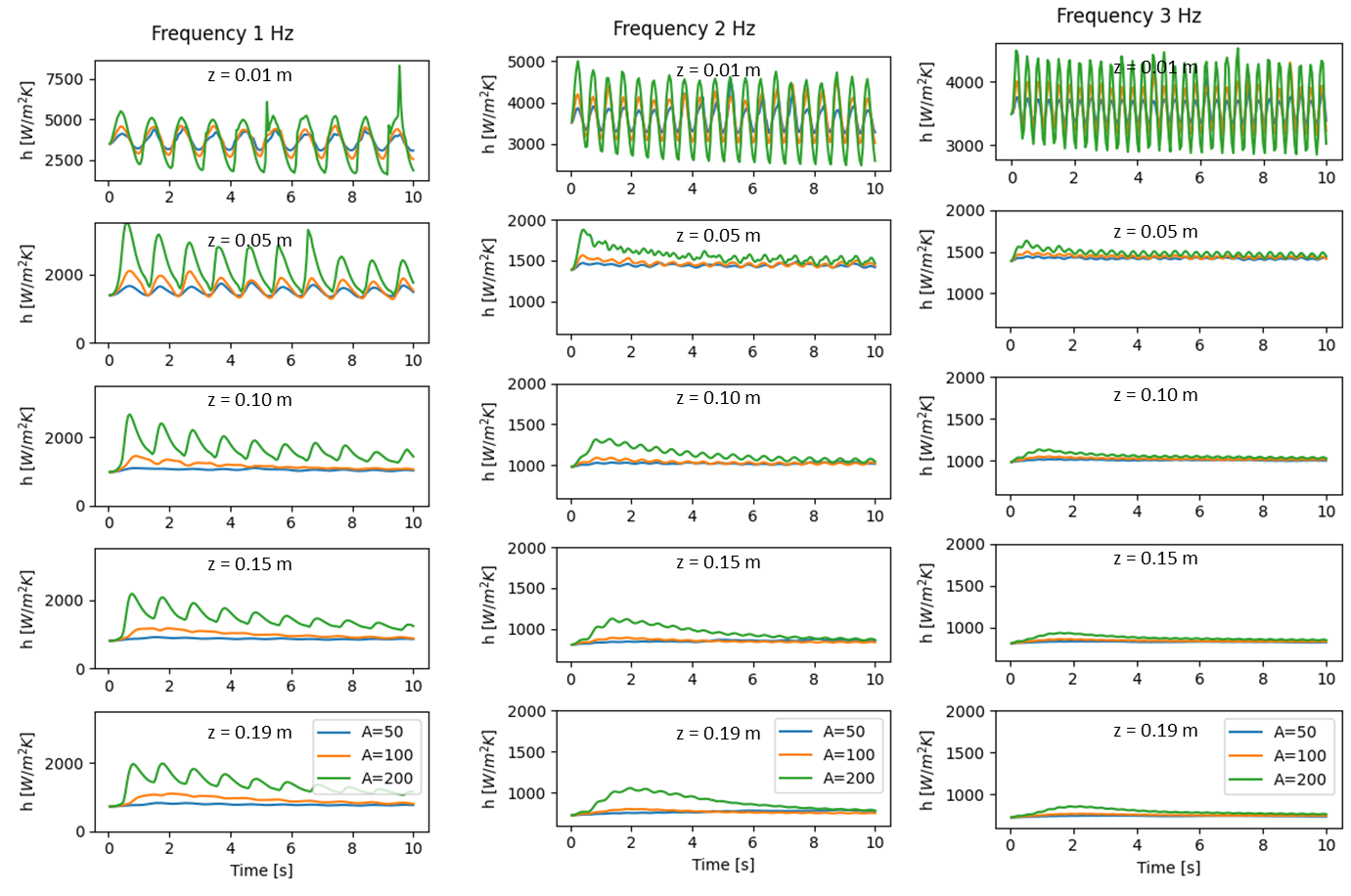


***Figure S5*** *Time dependent heat transfer coefficients along the aortic wall for the parametric sweep shown in Fig. S6 and Fig. 4. The average values in Fig. S6 and Fig. 4 were taken from the last 5 seconds.
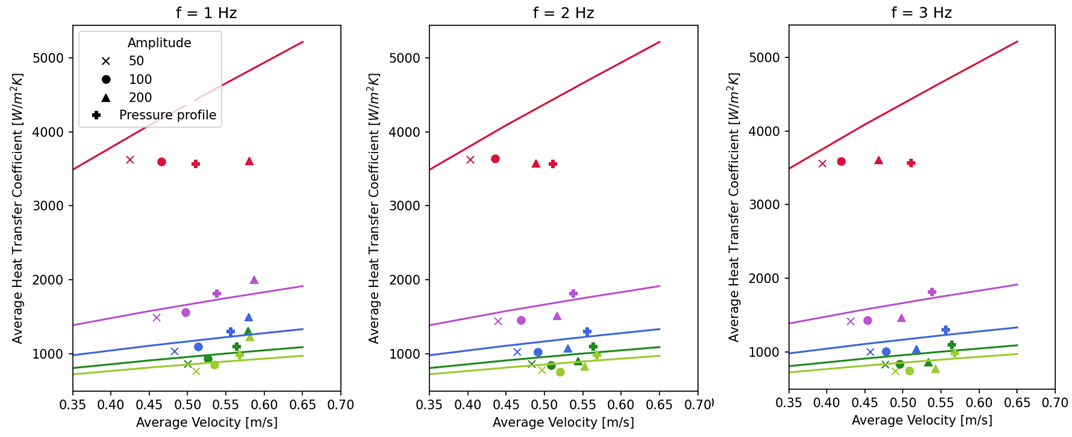
*

***Figure S6*** *Shows the heat transfer coefficients for* $f=1, 2, 3 \mathrm{Hz}$*in the sinusoidal parametric sweep. The heat transfer coefficient and the velocity were averaged over the last 5 seconds of the simulation. There is a clear tendency of decreasing heat transfer coefficient in relation to the constant velocity (continuously drawn line).*

***Table T3:*** *Percentage difference between the pulsatile flow and the stationary velocity for the sinusoidal simulations. The averaged percentage difference between the pulsatile element and the constant velocity for 1* Hz *is -1 ± 16* %*, for 2* Hz *is -10 ± 3* % *and for* 3 Hz *is -11 ± 2* %. *Specifically for A = 50*Pa/m *it is -9* %*, A = 100*Pa/m *it is -7* %*, and A = 200*Pa/m *it is 11* % *for 1*Hz. Further *for A = 50*Pa/m *it is -8* %*, A = 100*Pa/m *it is -11* %*, and A = 200*Pa/m *it is -9* % *for 2*Hz.

| $f=1 \mathrm{Hz}$ | | | | | $f=2 \mathrm{Hz}$ | | | | | $f=3 \mathrm{Hz}$ | | | | |
| --- | --- | --- | --- | --- | --- | --- | --- | --- | --- | --- | --- | --- | --- | --- |
| Distance  [m] | Amplitude  [Pa/m] | h­­­_1 Hz_  [W/m^2^K] | h­­­_stationary_  [W/m^2^K] | Difference  [%] | Distance  [m] | Amplitude  [Pa/m] | h­­­_1 Hz_  [W/m^2^K] | h­­­_stationary_  [W/m^2^K] | Difference  [%] | Distance  [m] | Amplitude  [Pa/m] | h­­­_1 Hz_  [W/m^2^K] | h­­­_stationary_  [W/m^2^K] | Difference  [%] |
| 0.01 | 50 | 3,622 | 3,930 | -8 | 0.01 | 50 | 3,626 | 3,901 | -7 | 0.01 | 50 | 3557 | 3751 | -5 |
|  | 100 | 3,598 | 4,172 | -14 |  | 100 | 3,640 | 3,996 | -9 |  | 100 | 3593 | 3900 | -8 |
|  | 200 | 3,610 | 4,815 | -25 |  | 200 | 3,574 | 4,305 | -17 |  | 200 | 3609 | 4189 | -14 |
| 0.05 | 50 | 1,490 | 1,592 | -6 | 0.05 | 50 | 1,441 | 1,555 | -7 | 0.05 | 50 | 1416 | 1541 | -8 |
|  | 100 | 1,564 | 1,660 | -6 |  | 100 | 1,452 | 1,610 | -10 |  | 100 | 1432 | 1583 | -10 |
|  | 200 | 1,999 | 1,812 | 10 |  | 200 | 1,510 | 1,691 | -11 |  | 200 | 1465 | 1660 | -12 |
| 0.10 | 50 | 1,035 | 1,146 | -10 | 0.10 | 50 | 1,024 | 1,125 | -9 | 0.10 | 50 | 1001 | 1116 | -10 |
|  | 100 | 1,096 | 1,181 | -7 |  | 100 | 1,022 | 1,156 | -12 |  | 100 | 1011 | 1140 | -11 |
|  | 200 | 1,498 | 1,256 | 20 |  | 200 | 1,074 | 1,202 | -11 |  | 200 | 1036 | 1186 | -13 |
| 0.15 | 50 | 857 | 956 | -10 | 0.15 | 50 | 861 | 941 | -9 | 0.15 | 50 | 826 | 934.6 | -12 |
|  | 100 | 930 | 980 | -5 |  | 100 | 842 | 964 | -13 |  | 100 | 833 | 53 | -13 |
|  | 200 | 1,305 | 1,028 | 27 |  | 200 | 899 | 997 | -10 |  | 200 | 857 | 987 | -13 |
| 0.19 | 50 | 765 | 863 | -11 | 0.19 | 50 | 779 | 851 | -8 | 0.19 | 50 | 739 | 846 | -13 |
|  | 100 | 855 | 883 | -3 |  | 100 | 755 | 871 | -13 |  | 100 | 746 | 861 | -13 |
|  | 200 | 1,225 | 919 | 33 |  | 200 | 824 | 897 | -8 |  | 200 | 772 | 889 | -13 |

***Table T4:*** *Percentage difference between the pressure profile interpolated from literature from an aortic pressure measurement and the stationary velocity. The average total percentage difference is 1 ± 12*%. *Not counting z = 0.01* m*, the average value is 7 ± 2* %.

|  | 0.01 m | 0.05 m | 0.10 m | 0.15 m | 0.19 m |
| --- | --- | --- | --- | --- | --- |
| h­­­_interpolated P_  [W/m^2^K] | 3562 | 1816 | 1298 | 1096 | 985 |
| h­­­_stationary_  [W/m^2^K] | 4432 | 1731 | 1231 | 1015 | 090 |
| Difference  [%] | -20 | 5 | 5 | 8 | 8 |

*
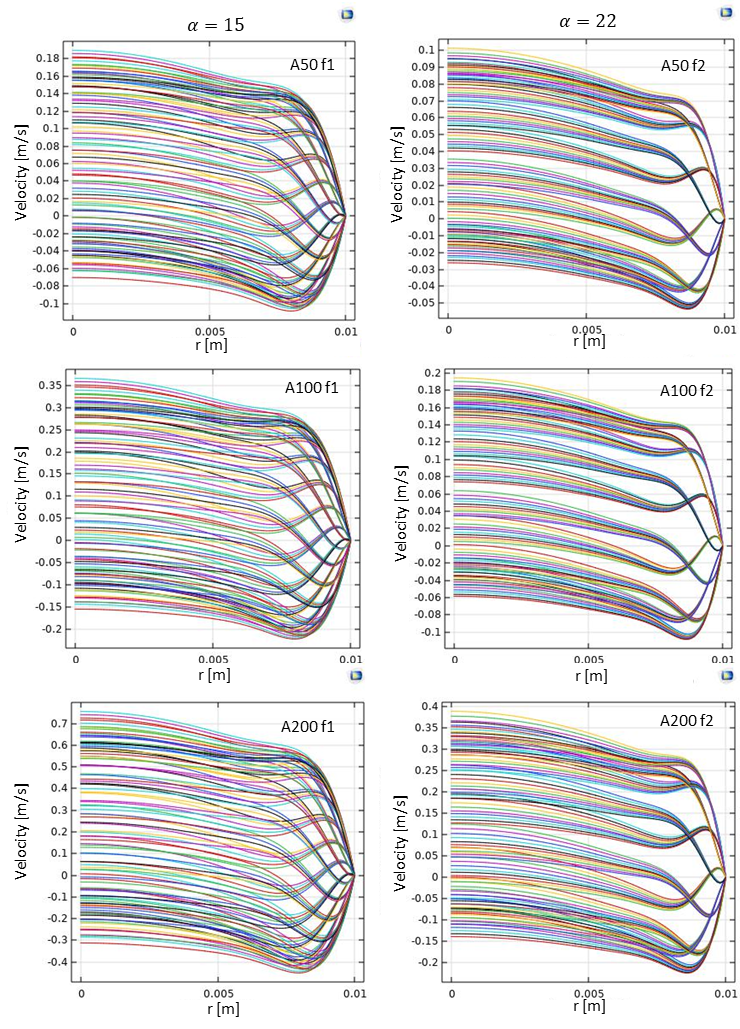
*

***Figure S7*** *Velocity profiles along the radial direction for the different swept parameters of A and f. The color code shows different times over the time periods.*

**5. Parameters swept**

***Table T5*** *Parameters swept in Fig. 6.*

| Infectious heat source [W/m^3^] | Heat transfer coefficient [W/m^2^K] | Infection radius [mm] | Distance between infection and sensor [mm] |
| --- | --- | --- | --- |
| 50 | 200 | 0.5 | 0 |
| 200 | 600 | 1 | 0.278 |
| 1,000 | 1,200 | 2 | 0.556 |
| 2,000 | 2,400 | 4 | 0.833 |
| 4,000 | 4,800 |  | 1.111 |
| 10,000 |  |  | 1.389 |

**6. Heat transfer efficiency upon a material parametric sweep**

A variation of the considered materials for the vascular graft material was used for the simulation (PDMS, PTFE, ePTFE, Dacron) as shown in the Table. PDMS is simulated as a possible quick prototyping material for an in vitro experimental test system used in the future. The wall thickness of a vascular graft is 1 mm, and the thickness of the sensor considered is 0.5 mm, which leaves 250 μm. As a variation, we also used a vascular graft thickness of 1.5 mm, which leaves 500 μm around the sensor. The heat transfer efficiency is dependent on the thermal conductivity, k (k_PDMS_ < k_PTFE_ < k_ePTFE_ < k_Dacron_). As a comparison, the physiological aortic wall thickness is around 2 mm ^5^ leaving 750 μm around the sensor. However, this simulation does not reflect the discussed physiological case of resectioning of aortic tissue with the replacement of a vascular graft. An increase in the heat transfer coefficient corresponding to an increase in heat transfer efficiency as shown in Figure S8. An increasing thickness of the material surrounding the heat flux sensor decreases the heat transfer efficiency.

***Table T6*** *The different material thermal properties used for the FEM simulation.*

| Property | PDMS | PTFE | ePTFE | Dacron (PETP) | Aorta |
| --- | --- | --- | --- | --- | --- |
| k [W/mK] | 0.16 | 0.2 | 0.35 | 0.51 | 0.46 |
| C_p_ [J/kgK] | 1460 | 960 | 2600 | 1300 | 960 |
| ρ [kg/m^3^] | 970 | 1230 | 1230 | 1380 | 2200 |

Increasing heat transfer coefficients increase the heat transfer efficiency of the system. In the case of an expanding vascular graft over time, the heat transfer efficiency will increase over time as shown in Fig. S8.


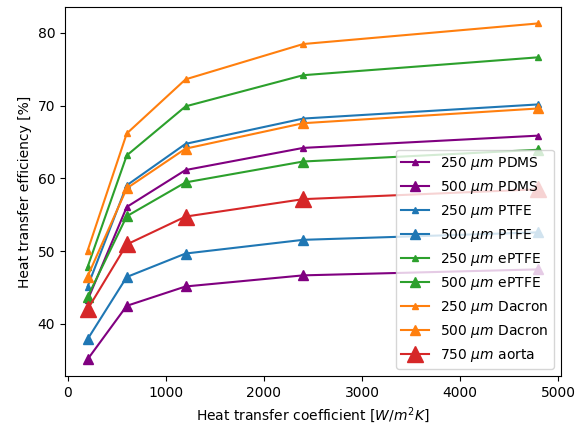


***Figure S8*** *Heat transfer efficiency of the aorta in a 3D heat transfer simulation for PDMS, PTFE, ePTFE, Dacron and aortic tissue.*

***Table T7*** *Difference in the heat transfer efficiency of the system between a heat transfer coefficient of* $200 W/m^{2}K$ *and* $4800 W/m^{2}K$*. This allows for an insight into the changes in the heat transfer coefficient along the aorta wall.*

| Material | Thickness | % at h=200 W/m^2^K | % at h=4800 W/m^2^K | % difference |
| --- | --- | --- | --- | --- |
| PDMS | 250 μm | 43 | 66 | 23 |
| PDMS | 500 μm | 35 | 47 | 12 |
| PTFE | 250 μm | 45 | 70 | 25 |
| PTFE | 500 μm | 38 | 53 | 15 |
| ePTFE | 250 μm | 48 | 77 | 29 |
| ePTFE | 500 μm | 44 | 64 | 20 |
| Dacron | 250 μm | 50 | 81 | 31 |
| Dacron | 500 μm | 47 | 70 | 23 |
| Aortic tissue | 750 μm | 42 | 59 | 17 |

**7. Temperature increase in the 3D model**


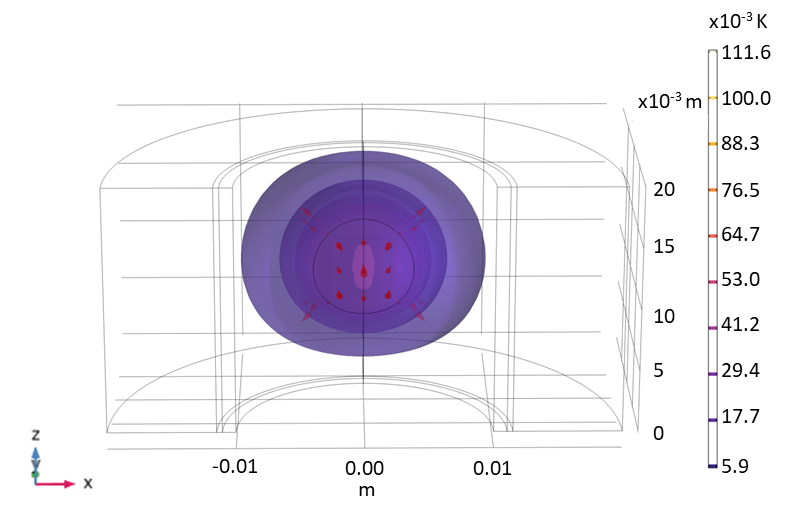


***Figure S9:*** *Temperature increase surrounding the infectious heat source.*

The boundary conditions of the simulation were set to 310.15 K (37°C) as the temperature increases even for the largest heat source and heat density only increased the temperature by 0.015 K as also shown in Fig. S10. We therefore assumed that the localized temperature increase is negligible, allowing for a set temperature boundary condition.


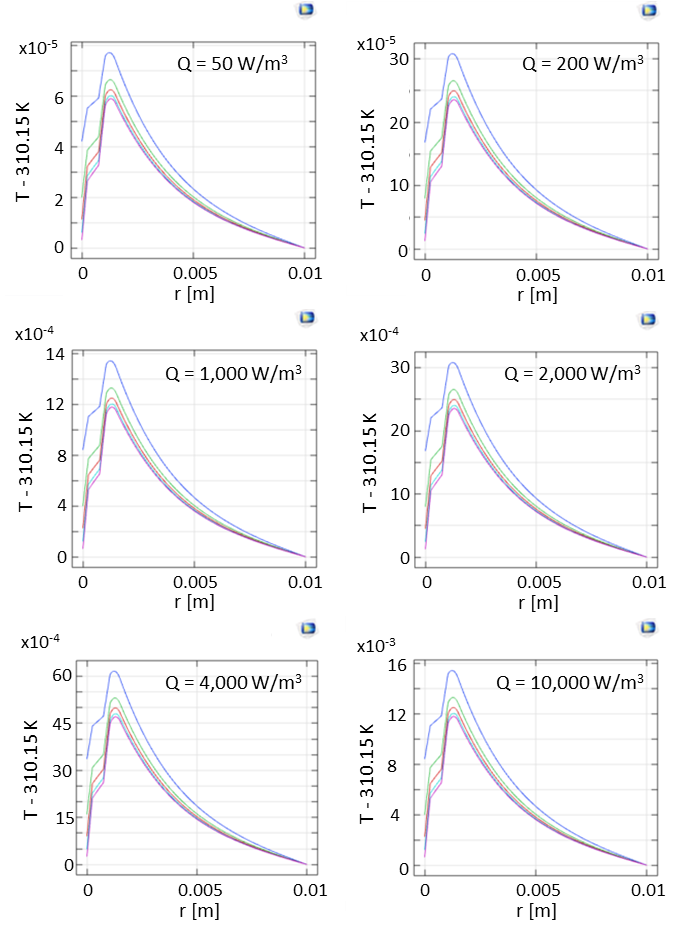


***Figure S10:*** *Temperature profile through sensor, infection and tissue for different infectious heat source thermal density, Q, each with increasing heat transfer coefficients, h (200, 600, 1,200, 2,400, and 4,800* W/m^2^K *are represented by the different colors throughout the figures). The temperature increases with increasing heat transfer coefficient.*

**8. Variation of the heat transfer coefficient depending on changes in radius**

*
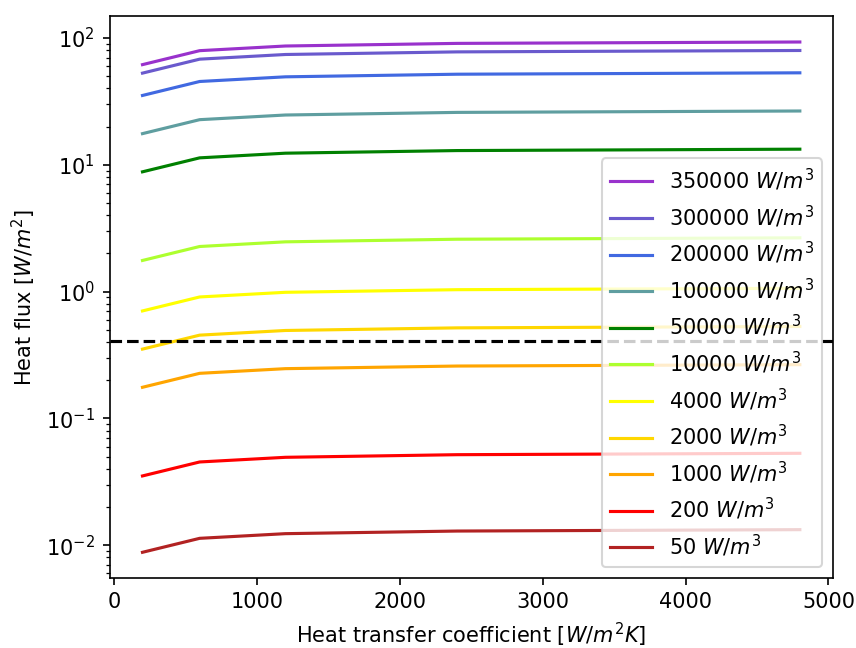
*

***Figure S11*** *Increasing the heat source of the infectious area for a radius of the infection of* $4 \mathrm{mm}$ *and a thickness of* $450 \mu m$*. The dotted line an example of the resolution of a commercial heat flux sensor (*$0.41\frac{W}{m^{2}K}$*).*

Fig. S11 shows the full sweep of the different infectious thermal densities Q [W/m^3^]. Above a Q of 2,000 W/m^3^, for the defined range of heat transfer coefficients, the heat flux through the sensor is above the resolution threshold of a commercial heat flux sensor (0.41 W/m^2^).

***Table T8*** *Heat sources and the respective number of bacteria in the case of an infection thickness of 450* μm *and radius of 4* mm*.*

| Heat source [$W/m^{3}$] | # bacteria | Comparison to biofilm [%] |
| --- | --- | --- |
| 50 | 3,231 | 0.01 |
| 200 | 12,925 | 0.06 |
| 1,000 | 64,627 | 0.29 |
| 2,000 | 129,254 | 0.57 |
| 4,000 | 258,508 | 1.14 |
| 10,000 | 646,270 | 2.86 |
| 50,000 | 3,231,352 | 14.29 |
| 100,000 | 6,462,705 | 28.57 |
| 200,000 | 12,925,409 | 57.14 |
| 300,000 | 19,388,114 | 85.71 |
| 350,000 | 22,618,467 | 100.00 |

The volume of a single bacterium has previously been determined as 1.49 μm^3^ ^6,7^ and the thermal power of a single *E. coli* is 3.5 pW ^7^, yielding a thermal density of a singular bacteria of 2,350,000 W/m^3^. Bacterial biofilms have a bacterial occupation of 15 % ^7^ yielding a thermal power of 350,000 W/m^3^. The thermal power of the heat sources is expressed as percentages in relation to the thermal power of a bacterial biofilm.

*
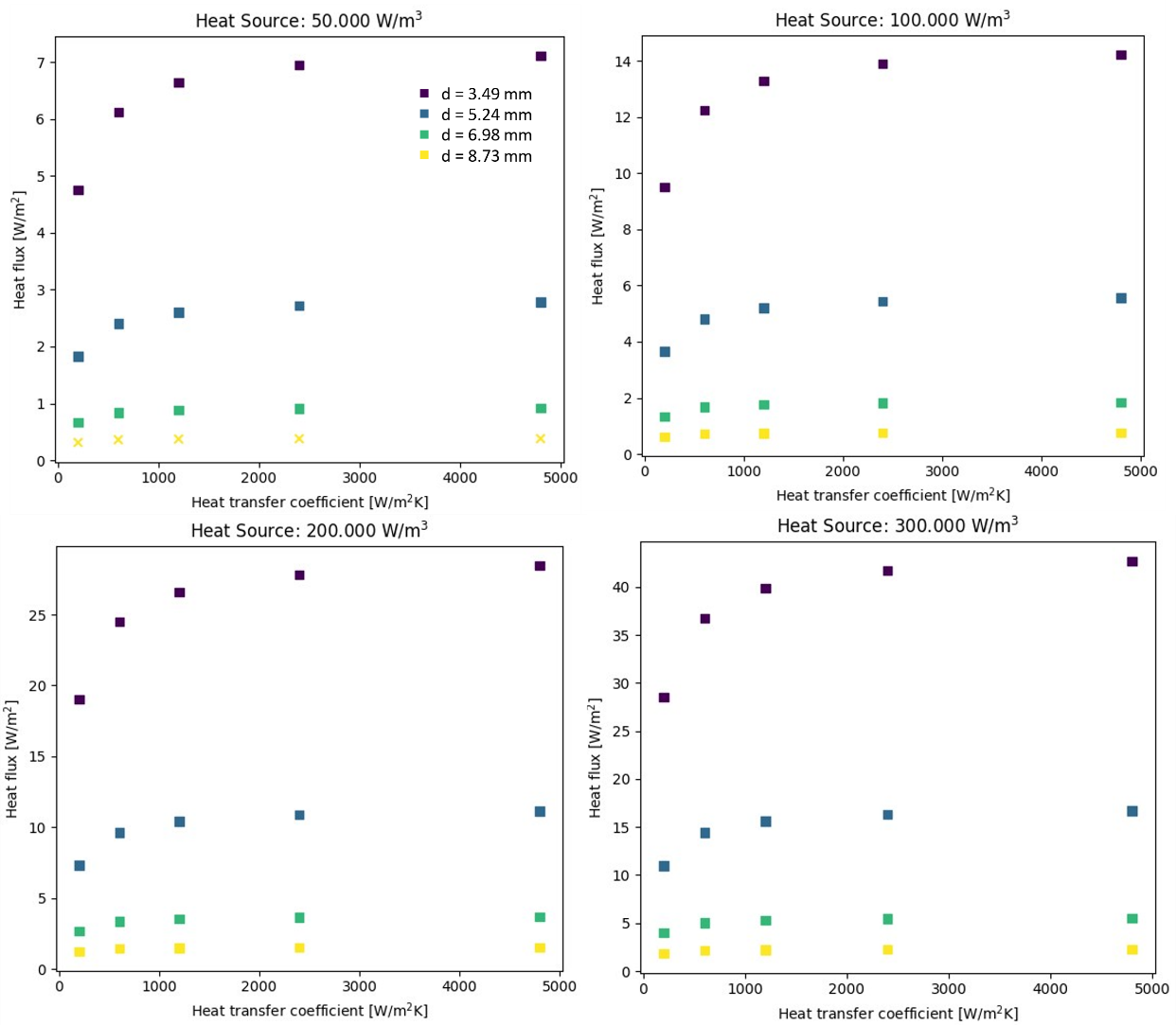
*

***Figure S12*** *Increasing the heat source at different distances to the infection for an infectious source of 4 mm radius and 450 μm thickness.*

**9. Simulation build and the calculation of arc length, d**

The build of the simulation is shown in Fig. S13. The arc length, d, is the distance between the center of the heat flux sensor to the center of the infection. The distance is varied by the arc angle, φ, and the arc length is determined as: d = r φ 2π/360.

*
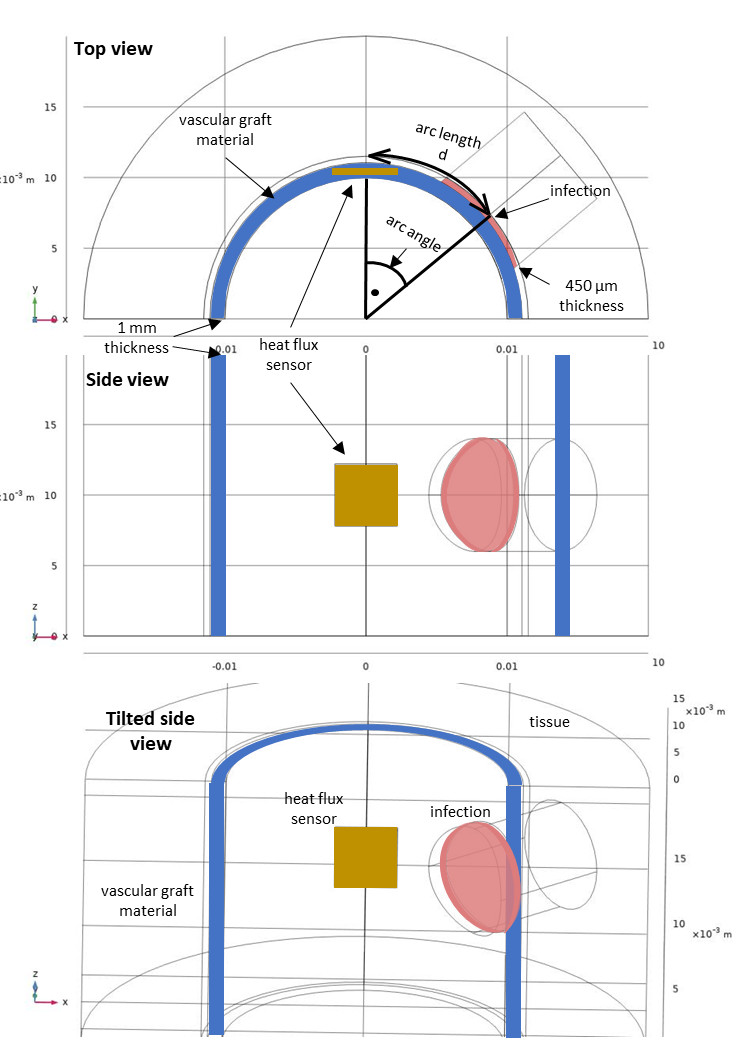
*

***Figure S13*** *Top view, side view and the tilted side view of the build of the vascular graft heat transfer simulation. The arc length, d, is determined through the arc angle* φ*.*

Given a radius of 10 mm and a graft length of 200 mm, the number of sensors can be calculated as the whole area of the cylinder divided by the unit cell area (as shown in Fig. S13):

$$N_{hexagonal}=\frac{graft surface area}{hexagonal unit cell area}=\frac{2\pi rh}{\frac{3\sqrt{3}}{2}d_{min}^{2}}=\frac{4\pi}{3\sqrt{3}}\frac{rh}{d_{min}^{2}}$$

With r as the graft radius, h as the total graft (graft) length, and d_min_ as the determined minimum distance between the center of the sensor and infection. This yields 177 sensors for d_min_ = 5.24 mm and 100 sensors for d_min_ = 6.98 mm.


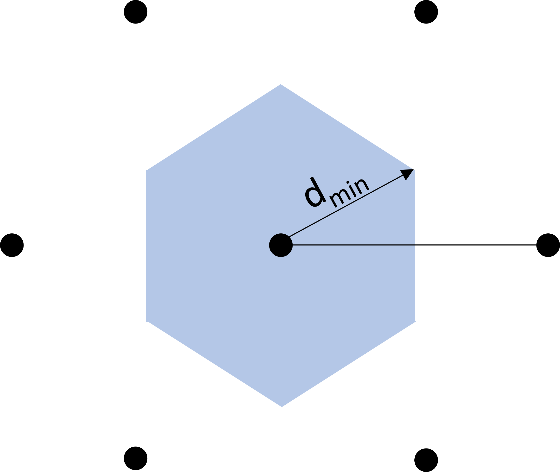


***Figure S14*** *Unit cell area of a hexagonal grid positioning of the sensors. The dark circles indicate the location of the sensor centers.*

**References**

1. Cengel, Y. A. *Heat Transference a Practical Approach (Second Edition)*. *McGraw-Hill* vol. 4 (2004).

2. Mersmann, A. *Thermische Verfahrenstechnik*. *Thermische Verfahrenstechnik* (Springer Berlin Heidelberg, 1980). doi:10.1007/978-3-662-13211-1.

3. Etz, C. D. *et al.* Vascular Graft Replacement of the Ascending and Descending Aorta: Do Dacron Grafts Grow? *Annals of Thoracic Surgery* **84**, 1206–1213 (2007).

4. Womersley, J. R. Method for the calculation of velocity, rate of flow and viscous drag in arteries when the pressure gradient is known. *J Physiol* **127**, 553–563 (1955).

5. Liu, C. Y. *et al.* Evolution of aortic wall thickness and stiffness with atherosclerosis: Long-term follow up from the multi-ethnic study of atherosclerosis. *Hypertension* **65**, 1015–1019 (2015).

6. Narisawa, N., Furukawa, S., Ogihara, H. & Yamasaki, M. Estimation of the Biofilm Formation of Escherichia coli K-12 by the Cell Number. *J Biosci Bioeng* **99**, 1–3 (2005).

7. Philipps, R., Kondev, J., Theriot, J. & Garcia, H. G. *Physical Biology of the Cell*. vol. Second Edition (Garland Science, 2013).
